# European identity and cultural heritage: the Mediterranean has its say

**DOI:** 10.12688/openreseurope.21507.1

**Published:** 2025-10-17

**Authors:** Sónia Bombico, Leonor Dias Garcia

**Affiliations:** 1 IIFA - Instituto de Investigação e Formação Avançada, Universidade de Evora Centro Interdisciplinar de Historia Culturas e Sociedades, Palácio do Vimioso, Largo do Marquês de Marialva, n.º 8, Évora, 7000-809, Portugal; 2 University of Évora Interdisciplinary Centre for History Cultures and Societies, Palácio do Vimioso, Largo do Marquês de Marialva, n.º 8, Évora, 7000-809, Portugal

**Keywords:** Cultural heritage; European identity; Mediterranean; European heritage policy; cultural dialogue

## Abstract

Mediterranean Cultural Heritage (MCH) has become increasingly prominent within European cultural policy and identity-building strategies. This article explores how MCH has been addressed in European initiatives and academic research, combining qualitative analysis of institutional and policy frameworks with bibliometric network analysis. The study focuses on three main areas: the role of the Union for the Mediterranean (UfM) in heritage-related actions; the presence of MCH in two key European initiatives — the European Heritage Label and the Cultural Routes of the Council of Europe; and the identification of trends in European academic production since the launch of the Barcelona Process in 1995. Findings show that MCH has been mobilised as a tool for cultural diplomacy, regional cooperation, and the promotion of shared values, although its representation often remains implicit and fragmented. While initiatives such as the Day of the Mediterranean and the Mediterranean Capitals of Culture and Dialogue highlight growing recognition of Mediterranean diversity, challenges persist concerning Eurocentrism, limited inclusivity, and instrumentalisation of heritage. Bibliometric analysis also reveals a strong focus on themes such as sustainability, climate change, and intangible heritage, with the Mediterranean Diet emerging as a particularly influential topic. The article concludes that MCH is increasingly used to support European identity narratives and regional engagement, yet calls for more integrated and participatory approaches that reflect the complexity of the Mediterranean space and its diverse cultural legacies.

## Plain language summary

This study explores how the cultural heritage of the Mediterranean region contributes to shaping a shared European identity. Mediterranean Cultural Heritage includes traditions, sites, and ways of life found across countries bordering the Mediterranean Sea.

The research examines how this heritage has been used in European policies and programmes, such as the Union for the Mediterranean, the European Heritage Label, and the Cultural Routes of the Council of Europe. It also looks at trends in academic research from the last 30 years using a method called bibliometric analysis, which helps identify patterns in published studies.

The findings show that Mediterranean heritage is increasingly used to promote values like diversity, dialogue, and sustainability. Initiatives such as the Day of the Mediterranean and the Mediterranean Capitals of Culture and Dialogue highlight its growing visibility. However, there are still challenges — the contributions of southern Mediterranean countries are often underrepresented, and heritage is sometimes simplified or used mainly for political purposes.

Overall, the study shows that Mediterranean heritage holds strong potential to bring people and cultures together. A more inclusive and long-term approach is needed to ensure that this rich and diverse legacy plays a meaningful role in building a European identity that reflects the region’s complexity.

## Introduction

The ongoing discourse surrounding European identity highlights the tension between cultural diversity and the notion of a unified European identity. The EU’s cultural policy reflects a growing emphasis on embracing diversity while maintaining a framework of shared values. This nuanced approach acknowledges the complexity of European identity, shaped by both common histories and distinct cultural expressions.

Cultural heritage plays a pivotal role in defining collective identity and memory, linking local artifacts to the broader human community. This dual function – local and universal – is essential in the geopolitical processes that shape nation-states and the international community ( Isnart, 2012). Within this framework, the Mediterranean cultural heritage stands out, offering a rich blend of traditions that embody both shared European experiences and regional diversity. The Mediterranean region's heritage is a testament to its historical interactions and shared cultural expressions, contributing to the broader European identity ( Bullen & Isnart, 2020).

Recently, the European Union has increasingly leveraged cultural heritage as a strategic tool, not only to foster cohesion among member states but also to strengthen ties with non-EU countries. These initiatives aim to cultivate a shared European identity, support integration, and, more recently, address global challenges by using culture to promote dialogue and cooperation beyond EU borders ( Lahdesmaki *et al.*, 2020).

Considering this framework, in this article we aim to illustrate how Mediterranean Cultural Heritage (MCH) has been used, promoted, and studied within European initiatives and policies for the Mediterranean region. The analysis is organised into the following stages:

a) Analysis of the UfM (Union for the Mediterranean) action, based on documents such as the official website, work programmes, and reports from the last 10 years (2015–2025). The goal is to understand how cultural heritage has been addressed and to identify the main areas of focus for this intergovernmental institution.
b) Analysis of the representativeness and role of MCH within key European initiatives promoting European cultural heritage and identity. The analysis focused on two major initiatives – the European Heritage Label (EHL) and the Cultural Routes of the Council of Europe. This analysis aimed to clarify the position of MCH within broader European heritage policies.
c) Identification of key trends in European academic research agendas, based on the results of scientific production over the past 30 years (since the start of the Barcelona Process in 1995). This analysis was carried out using Bibliometric Network Analysis, a quantitative methodology that helped identify emerging patterns and trends.

The combination of a qualitative approach – focused on the analysis of political and institutional initiatives – with a quantitative methodology based on bibliometric analysis aims to identify trends and gaps in how Mediterranean Cultural Heritage has been addressed in scientific research. The present work also seeks to analyse how it has been valued within European cultural heritage policies, how it has been used in cultural diplomacy in the Euro-Mediterranean region – in connection with the evolution of European agendas for the region – and how it is contributing to the promotion of European identity.

## The Idea of the Mediterranean

Over the past decades, the Mediterranean has been the subject of intense historiographical, geographical, anthropological, and literary reflection. More than a defined geographical unit, this space is conceived as a complex constellation of human interactions, where the sea functions both as a medium and a metaphor.

The key contributions that have shaped the study of the Mediterranean, emphasizing its diverse epistemological, methodological, and symbolic frameworks.

The foundational work of Fernand Braudel (1949) about the mediterranean world represents a landmark in the renewal of historiography. Braudel introduced a layered temporal reading – the concept of the “longue durée” – in which geographical and socio-economic factors take precedence over episodic political events. His structural approach situates the Mediterranean as a space of permanence and slow rhythms, where landscape, climate, and daily practices shape ways of life as much as empires or battles.

Braudel’s work laid the foundation for viewing the Mediterranean as a unified historical space ( Bicchi, 2022; Weitzman, 2012).

This structural focus, however, contrasts with more fragmented readings, attentive to the cultural and affective diversity of the Mediterranean space, such as that of Predrag Matvejević (1987). The author offers an essayistic and poetic approach, in which the Mediterranean is constructed from its shores, its languages, and its objects. His perspective is marked by fragmentation and plurality, rejecting the notion of a homogeneous Mediterranean identity and instead proposing an affective and subjective topography. In the *Mediterranean Breviary* ( 1987), Matvejević offers a nuanced view of Mediterranean identity as a “satura” of differences and conflicts, rather than a unified whole ( Botta, 2010).

Paul Balta also underlined the ambivalent character of the Mediterranean space – simultaneously a stage for civilisational encounters and historical confrontations. His geopolitical analysis reinforces the interpretation of the Mediterranean as a border zone, where religious, cultural, and economic affiliations intersect, often in tension ( Balta, 1997).

This complexity is further deepened in the work of Horden and Purcell (2000), which marks a new paradigm in Mediterranean history by proposing an approach centred on micro-scale and historical ecology. The Mediterranean is conceived here as an “ecology of micro-regions”, interconnected through networks of mobility and adaptation, rather than by grand political or civilisational unities. This model values local diversity and interconnectivity as key interpretative tools. Horden and Purcell emphasize the connectivity and ecological unity of the Mediterranean region, highlighting the shared cultural practices and social structures that transcend local differences.

In a similarly critical vein, William V. Harris (2005) calls for a deconstruction of the idea of a unified Mediterranean civilization. His work emphasises temporal and spatial discontinuities and asymmetries, suggesting that the category “Mediterranean” should be problematised as a historiographical construction rather than taken as a mere geographical given.

David Abulafia (2011) proposes a return to narrative, without abandoning complexity. Through a diachronic analysis that privileges human interactions – commercial, political, religious, and cultural – Abulafia presents the Mediterranean as a dynamic and fluid space, criss-crossed by maritime routes that facilitate the movement and exchange of people, ideas, and goods.

Complementary, the *Companion to Mediterranean History*, edited by Horden and Kinoshita (2014), consolidates a multidisciplinary approach to the Mediterranean, bringing together contributions from history, anthropology, archaeology, and cultural studies. This work explores the study of Mediterranean space, emphasizing the significance of networks, scales, and mobilities.

In the Portuguese context, Orlando Ribeiro (1982) offers a crucial geo-cultural reflection for understanding Portugal’s role as a bridge between the Atlantic and the Mediterranean. His analysis of territory and civilisation highlights how southern Portugal shares many Mediterranean characteristics, without fully identifying with the region. This represents a partial, yet significant, affiliation, with implications for cultural identity, spatial organization, and social practices.

More recently, Filipe Themudo Barata (2024) proposes a personal and essayistic perspective, in which the Mediterranean is simultaneously memory and subjective construction. By interweaving experiences, landscapes, and readings, the author evokes an affective geography that reinforces the idea of the Mediterranean as a space of cultural projection and identity introspection.

As we have seen, it is equally essential to consider the contributions of literature and philosophy. In this regard, the work of Albert Camus (1913–1960) envisions the Mediterranean cultural Renascence as both a unifying force between Western and Eastern traditions and an alternative path for Europe – one that rejects the destructive ideologies of Fascism and Russian Collectivism. By embracing a Mediterranean identity rooted in diversity and shared human values, he advocates for a more humane, inclusive, and peaceful future for European civilisation. This so-called “Third Way” embodies his humanism, promoting a vision of society that prioritises diversity and dialogue over conflict and division ( Davison, 2000) – an idea that remains strikingly relevant in contemporary Europe, as it grapples with rising populism, cultural polarisation, and the ongoing search for a cohesive yet plural identity.

In summary, studies on the Mediterranean bring together a variety of perspectives, ranging from unifying and structuralist models to more critical, fragmented, and interdisciplinary approaches. Today, the Mediterranean is understood not only as a physical or historical space, but also as an epistemological and symbolic field. This multiplicity of perspectives does not weaken the concept – rather, it enriches it, making it more capable of reflecting the complexity of this region’s past and present.

Accordingly, the study of Mediterranean identity is a rich and diverse field, encompassing a wide range of perspectives and approaches. From the historical and cultural constructions of Braudel, Horden and Purcell, to the literary and philosophical insights of Matvejević, the Mediterranean has emerged as a complex and multifaceted region. The socio-political implications of Mediterranean identity, particularly in the context of migration and otherness, highlight the need for a nuanced and critical approach to the study of this region. As the Mediterranean continues to evolve in the context of globalization and postcolonialism, the study of Mediterranean identity remains a vital and dynamic field of research.

The concept of “mediterraneanity” (or “mediterranean identity”) is multifaceted, encompassing cultural, historical, geographical, climatic, and socio-economic dimensions. While often used to describe the distinctive characteristics of the Mediterranean region, its scope extends beyond geography to include cultural and ideological constructions. As such, Mediterranean identity represents a dynamic and evolving construct, shaped by centuries of historical interaction, cultural exchange, and contemporary political realities.

Historically, the Mediterranean has served as a crossroads between Christian (including Roman European) and Islamic (Arab) civilisations, acting as a historic bridge for a variety of ethnic, religious and cultural traditions – thereby contributing to a regional identity not constrained by strict national boundaries ( Affaya, 2009). This intermingling has fostered a sense of both unity and diversity, requiring careful negotiation and dialogue in a globalised world.

Nevertheless, “mediterraneanity” was also co opted as an ideological instrument by fascist Italy. Between 1935 and 1940, it was deployed to legitimise imperial ambitions and cultural assimilation – particularly in the “realised” colonies such as North and East Africa. In these contexts, Mediterranean identity was portrayed as rooted in a shared Roman Latin heritage, thereby marginalising the complex realities and cultural diversity of local populations, who were often omitted from this narrative ( Epolito, 2015).

Despite its symbolic potential, the notion of a unified Mediterranean identity often encounters challenges. Several scholars highlight the risk of reinforcing old stereotypes through Mediterranean studies, a phenomenon referred to as “mediterraneism” – the tendency to romanticise or oversimplify the region, thereby neglecting its internal complexities ( Botta, 2010; Herzfeld, 2020). Such critiques highlight the need to recognise the Mediterranean’s plurality and to view it also as a space of tensions, differences, and conflicts.

Sociologist Gerhard Steingress presented a critical and theoretically grounded reflection on the concept of “mediterraneanity” as cultural heritage. He defines it as a historically produced, complex cultural medium expressed through dense networks of social, cultural, and symbolic interaction across the Mediterranean. Rejecting the notion of a fixed Mediterranean identity, Steingress frames “mediterraneanity” as a dynamic construct shaped by processes of hybridization, acculturation, and transculturation. He describes it as *“the living culture of the Mediterranean”*, advocating for a view of heritage as a means of cultural communication, coexistence, and peace, rather than a static identity marker ( Steingress, 2005).

At the policy level, the concept of “Mediterraneanity” has indeed shaped European frameworks of regional cooperation and identity-building. Yet, attempts at Euro Mediterranean integration have often faltered due to a reliance on geographical myth rather than recognition of the region’s diverse political, cultural and economic dynamics ( Moisseron & Bayoumi, 2012). These tensions are particularly visible in debates surrounding cultural heritage, national identity, and regional cohesion.

Cultural heritage plays a central role in this discourse: it is both a marker of regional pride and a universal human asset. The UNESCO designation of the Mediterranean diet symbolises this shared cultural heritage, while also highlighting the challenges of preserving such heritage in a region marked by historical vulnerabilities and political instability ( Lowenthal, 2008; Scepi & Petrillo, 2015). As such, the preservation and interpretation of Mediterranean heritage require not only technical measures but also critical engagement with identity politics and collective memory.

## The political instrumentalization of cultural heritage

The instrumentalization of cultural heritage is a widespread phenomenon, often serving political, economic, or social agendas beyond its intrinsic value. This dynamic can be seen across diverse contexts, where heritage becomes a tool for legitimacy, development, or cohesion. European cultural policies are no exception, frequently mobilising heritage narratives to promote regional identity, soft power, and socio-economic objectives.

Françoise Choay (1992) argued that heritage is not simply what we inherit, but what we choose to preserve – and such choices are inherently political, ideological, and cultural. She offers a critical framework for understanding how and why heritage is instrumentalised. Heritage, according to Choay, is a modern invention: it is neither eternal nor universal, but emerges in the modern western world, particularly from the eighteenth century onwards. From this point, the past begins to be preserved not for its practical use, but for its symbolic and identity value. Throughout the twentieth century, Choay warned, heritage had increasingly become an ideology. Detached from its original context, it was and is often transformed into an object of cultural veneration and mobilised politically – to legitimise national identities, exclude uncomfortable memories, or establish an “official” version of history. Yet, provided it does not become dogmatic, heritage can also serve as a space for dialogue, democratic memory, and critical engagement. Choay also highlighted the risks posed by the industrialisation of cultural heritage, particularly through the influence of cultural marketing and mass tourism.

These issues are especially pressing in the Mediterranean context — a region that concentrates a significant share of UNESCO World Heritage sites and remains the world’s leading tourist destination. In such a setting, the tension between preservation and commodification becomes particularly acute, raising fundamental questions about the future of heritage management.

A wide range of studies has addressed the political uses of cultural heritage ( Amineddoleh, 2020; Franceschini, 2024; Smith, 2006). These contributions underscore the extent to which cultural heritage is instrumentalised, whether to legitimise political authority, construct collective identities, or negotiate competing values.

A broad review of the literature shows that cultural heritage is often instrumentalised in recurrent ways: the construction of national narratives that erase episodes of foreign rule or external influence; its use as propaganda, particularly in totalitarian regimes; its mobilisation in territorial and ethnic conflicts through the deliberate destruction of monuments; ideologically driven reconstructions that reshape the past to suit present needs; and the strategic use of UNESCO or other international listing mechanisms to bolster sovereignty, prestige, or collective identities – a trend also visible in Europe and the Mediterranean, which is the focus of this article.

In the Mediterranean context, historical myths such as that of al-Andalus are reinterpreted to serve contemporary political agendas and to shape cultural and identity policies in the region ( Daguzan, 2016).

At the European level, cultural heritage has also been instrumentalised, with the European Union actively constructing a sense of shared heritage through a range of flagship initiatives: the European Capital of Culture and the European Heritage Days, both launched in 1985; the European Heritage Awards, awarded by Europa Nostra since 2002; the European Heritage Label, with its first designations in 2013; and the House of European History, inaugurated in 2017.

If we look at one of the oldest initiatives – the European Capital of Culture – it is interesting to note that these have served as a powerful vehicle for promoting Mediterranean heritage itself. Since 1985, a notable number of Mediterranean cities have held this title, including Athens (1985), Florence (1986), Thessaloniki (1997), Genoa (2004), Istanbul (2010), Marseille (2013), Matera (2019), Rijeka (2020), Paphos (2017), Valletta (2018), Eleusis (2023), and the cross-border twin cities of Nova Gorica/Gorizia (2025) – bringing the total to 12 Mediterranean cities to date.

The designation of 2018 as the European Year of Cultural Heritage (EYCH) illustrates how the EU mobilises heritage as a source of self-confidence and as a political tool to foster unity and trust in the future, particularly at a time when broader narratives of European renewal have failed to take hold ( Helly, 2018). The New European Agenda for Culture, launched in 2018 during the EYCH, marked a strategic shift in EU cultural policy ( European Commission, 2018). It redefined culture not just as a historical asset, but as a tool to tackle social and economic challenges. The agenda focused on three priorities: promoting social cohesion and well-being through cultural participation; boosting innovation and job creation in the cultural and creative sectors, especially via digital transformation; and strengthening international ties through cultural diplomacy.

Overall, 2018 marked a turning point, ushering in a more proactive and strategic cultural policy for a more inclusive and globally engaged Europe.

This trajectory builds on Article 128 of the Maastricht Treaty (1992), which for the first time articulated the notion of a European cultural heritage extending beyond national and regional traditions, and it has since been reinforced in the Union’s constitutional framework through the Treaty of Lisbon (2007). Here, the most recent discourse that frames culture and cultural heritage as key assets of the European project was formally consolidated: Article 3(3) of the Treaty on the European Union (2016) stipulates that the Union “shall respect its rich cultural and linguistic diversity and shall ensure that Europe’s cultural heritage is safeguarded and enhanced”.

In the field of European studies, particular attention has been given to the instrumentalization of cultural heritage as a strategy to reinforce European identity. Governments and political actors have long mobilised heritage to promote ideologies and to construct national or regional narratives. Heritage, in this sense, becomes a powerful tool for shaping or consolidating collective identities. A clear example can be found in Croatia, where the UNESCO World Intangible Cultural Heritage programme has been used to promote nation-building and to overcome past cultural suppression, thereby highlighting the role of heritage in constructing national identity and legitimising European aspirations ( Melis *et al.*, 2022).

At the European level, the deliberate mobilisation of cultural heritage, aligned with the established practices of EU institutions, is now regarded as increasingly important in countering the disintegrative forces that threaten the European project ( Jakubowski *et al.*, 2019). Yet while heritage can serve integrative and political ends, it is also essential to reflect on its potential drawbacks and ethical implications. The use of cultural heritage for political or economic purposes may lead to commodification, the erosion of authenticity, and the marginalisation of communities. At the same time, when managed inclusively and ethically, it can foster preservation, stimulate economic development, and enhance cultural sustainability. The balance between instrumental and intrinsic values thus remains a central and ongoing debate in cultural policy and heritage management.

Within this debate, a process of heritage making centred on the notion of a Mediterranean cultural heritage has emerged. Often framed in terms of unity, this process has been criticised for producing oversimplified or homogenised visions of the region. The Mediterranean Diet, inscribed by UNESCO as Intangible Cultural Heritage, stands as one of the most evident examples of such heritage-making.

At the same time, the political appropriation of Mediterranean cultural heritage has also been framed to strengthen European identity and to counter the forces of disintegration affecting the European Neighbourhood Policy, particularly with countries on the southern shore of the Mediterranean. The aim has been to construct regional and transregional narratives that resist discourses fuelling cultural and religious divisions, which risk fostering intolerance and the marginalisation of minorities. These challenges are further compounded by humanitarian crises in neighbouring regions, armed conflicts, terrorism, and economic pressures – all of which contribute to migration dynamics that the European Union has struggled to manage effectively.

Against this backdrop, the history of the Mediterranean and its cultural heritage present themselves as valuable resources for cultural dialogue, diplomacy, social inclusion, regional cooperation, and ultimately for peace. We contend that Mediterranean cultural heritage, in all its diversity and multicultural character, has in recent decades become deeply intertwined with European Union policies for the Euro-Mediterranean region, framed within a logic of constructing regional and macro-regional narratives of intercultural dialogue. This is the issue we now turn to: examining how this process of patrimonialisation has unfolded within the main Euro-Mediterranean institutions and in the context of European policies and initiatives on cultural heritage.

### The paradigmatic case of the Mediterranean Diet

The Mediterranean Diet is perhaps the most emblematic example of Mediterranean cultural heritage that comes to mind. It is a paradigmatic case worthy of close examination. Originally promoted by Ancel Keys in the mid-20th century as a healthy dietary model aimed at North American audiences, it was later reframed as intangible cultural heritage through a top-down process driven by Euro-Mediterranean political and institutional elites, culminating in its inscription on UNESCO’s Representative List of the Intangible Cultural Heritage of Humanity.

This shift marked a profound transformation: from a health-oriented nutritional model to a complex cultural and social construct. Today, the Mediterranean Diet embodies not only nutritional principles, but also social, cultural, and environmental dimensions – a living expression of Mediterranean identity that manifests in daily practices and broader societal values, and representing a harmonious relationship between people and their environment that contributes to a sense of identity and continuity ( Medina, 2021; Serra-Majem & Medina, 2020).

UNESCO’s recognition in 2010 (and its extension to three additional countries in 2013) formalised this multidimensional view. According to the nomination file, the Mediterranean Diet encompasses domains such as oral traditions, rituals, traditional ecological knowledge, and craftsmanship – thus fulfilling nearly all the criteria set out in the UNESCO Convention.

The institutional journey that led to the recognition of the Mediterranean Diet as Intangible Cultural Heritage by UNESCO began in 2005, during the Third Euro-Mediterranean Forum held in Rome. This event, titled *Dialogues between Civilisations and Peoples of the Mediterranean: The Food Cultures*, hosted the first preparatory meeting and culminated in the Declaration of Rome, where the idea of framing the Mediterranean Diet as cultural heritage was officially proposed for the first time.

In October 2007, the International Scientific Committee of the Mediterranean Diet Foundation convened in Barcelona, issuing the Barcelona Declaration, which formally advocated for the recognition of the Mediterranean Diet as intangible cultural heritage ( Reguant-Aleix *et al.*, 2009). This declaration was significant in reinforcing a broader vision of the diet – not merely as a nutritional model, but as a way of life deeply embedded in Mediterranean societies.

The process gained political momentum in December 2007, when Spain’s Ministry of Agriculture, Fisheries and Food hosted an international meeting in Madrid, bringing together representatives from Greece, Italy, and Morocco. The outcome was a joint commitment to submit a transnational application to UNESCO.

By 2008, the four countries had formalised the application. One of the key points of discussion at this stage concerned the terminology of the candidacy: whether to adopt the label *Mediterranean Diet*, *Mediterranean Traditional Diet*, or *Mediterranean Food*. Although there were differing views – particularly among anthropologists and cultural scholars who favoured more inclusive terms – the established public recognition of *Mediterranean Diet* ultimately prevailed.

In 2010, the Mediterranean Diet was inscribed on UNESCO’s Representative List of the Intangible Cultural Heritage of Humanity, with Spain, Greece, Italy, and Morocco as the initial submitting countries. The heritage community expanded further in 2013, with the inclusion of Portugal, Cyprus, and Croatia.

Following its patrimonialisation, the Mediterranean Diet entered a new phase under the guidance of the FAO and CIHEAM, recast as a sustainable food system ( Medina, 2021). The Mediterranean Diet thus became a symbolic vehicle for advancing sustainability discourses in the region, while still maintaining its core nutritional appeal.

Despite its institutional success, the Mediterranean Diet as heritage has not escaped critique. The anthropologist Michael Herzfeld has been especially sceptical, arguing that the Mediterranean Diet is largely a postmodern invention – a strategic cultural construct assembled to meet contemporary identity needs rather than reflect a truly shared historical food culture ( Herzfeld, 2014). Other authors argues that the Mediterranean Diet, as recognised today, is less a reflection of shared food practices and more a symbolic construction shaped by institutional, political, and scientific discourses — often disconnected from the actual dietary realities of Mediterranean populations ( Silva, 2016).

## Mediterranean cultural heritage in the context of European policies for the Euro-Mediterranean region

The evolution of Euro-Mediterranean relations has followed a progressive path from fragmented bilateral trade agreements in the 1960s and 70s to more structured and comprehensive cooperation frameworks. The Global Mediterranean Policy (1972–1992) marked the first coordinated approach, followed by the Renewed Mediterranean Policy in the 1990s, which introduced regional programmes and civil society engagement. A major turning point came with the launch of the Barcelona Process in 1995, establishing the Euro-Mediterranean Partnership (EMP), which integrated political, economic, and socio-cultural cooperation. In the 2000s, the European Neighbourhood Policy (ENP) introduced bilateral action plans and a conditionality-based approach. Finally, the Union for the Mediterranean (UfM), created in 2008, aimed to enhance political balance, institutional visibility, and project-based cooperation, while maintaining core objectives such as regional stability, integration, and development ( Albinyana, 2016).

The Union for the Mediterranean was established as an expansion and reformulation of the Barcelona Process, with the objective of addressing its limitations and enhancing regional cooperation between the European Union and Mediterranean countries ( Elistania *et al.*, 2019).

The Barcelona Process and its successor, the Union for the Mediterranean (UfM), represent major milestones in Euro-Mediterranean cultural politics, aimed at strengthening cooperation and integration between EU member states and Mediterranean countries. Conceived as responses to political, economic, and social challenges in the region, these frameworks sought to promote peace, stability, and prosperity through enhanced collaboration.

The Barcelona Declaration of 1995 explicitly highlighted the cultural dimension of this partnership, affirming that “the participants recognise that traditions of culture and civilisation throughout the Mediterranean region, dialogue between these cultures and the exchanges at human, scientific and technological level are an essential factor in bringing their peoples closer, promoting understanding between them and improving their perception of each other.”

Since then, cultural cooperation has been consistently presented as a pillar of the EU’s external action in the Mediterranean. Yet the treatment of cultural heritage has revealed a persistent tension between these declared ambitions and their practical implementation, with cultural heritage often addressed in vague, instrumental terms rather than through coherent, long-term strategies. The Association Agreements (1995–2006) signed with Tunisia, Morocco, Israel, Egypt, Jordan, Lebanon, Algeria and the Palestinian Authority contained only vague cultural clauses, focused largely on the audiovisual sector, while treating heritage in a marginal and instrumental manner, without the robust cultural cooperation protocols applied in other regions. In parallel, programmes such as *Culture 2000* promoted initiatives of cultural diplomacy, including the restoration of the city of Alexandria, which symbolically reinforced the idea of shared heritage but lacked long-term strategic continuity. The most consistent effort came with the *Euromed Heritage* programme (1998–2012), which over four phases evolved from inventories and institutional capacity-building to the valorisation of intangible heritage, seeking to implement the 2003 UNESCO Convention and culminating in an emphasis on social appropriation and heritage education, in line with the Faro Convention (2005). However, many projects suffered from administrative shortcomings and insufficient participation. Moreover, the issue of Mediterranean migration highlighted a further contradiction: while the EU actively supports the protection of heritage in conflict zones, it has failed to recognise or integrate the intangible cultural heritage of migrants within its own territory, undermining social inclusion and reinforcing dynamics of exclusion ( Jakubowski *et al.*, 2019).

Several authors argue that Euro-Mediterranean policies continue to be hindered by structural asymmetries, implementation challenges, and enduring geopolitical constraints ( Albinyana, 2016; Daguzan, 2016; Elistania *et al.*, 2019; Helly, 2018). They call for a more coherent, equitable, and context-sensitive approach, particularly in relation to the southern Mediterranean.

Damien Helly (2018) highlights a persistent disconnect between the stated objectives and the practical implementation of cultural policies, noting a clear gap between the rhetoric of shared values and cultural cooperation and the realities on the ground. He contends that these policies often suffer from a Eurocentric bias, as they fail to adequately reflect the perspectives and needs of countries in the southern Mediterranean. Moreover, Helly underscores the limited involvement of civil society and non-state actors in both the design and execution of such initiatives, a shortcoming that ultimately undermines their legitimacy and effectiveness.

Research on Euro-Mediterranean relations reflects similar imbalances. Scientific collaboration in this field remains strongly Eurocentric, with contributions from scholars based in the southern Mediterranean still significantly underrepresented ( Kourtelis, 2024). The same author notes that academic interest in the region has fluctuated: it grew with the launch of the UfM in 2008 and the Arab uprisings of 2010, was renewed in 2014–2015 following the second revision of the European Neighbourhood Policy (ENP), but declined sharply after 2016, mirroring reduced EU political commitment and stalled reforms in most Mediterranean countries ( Kourtelis, 2024).

EU funding programmes are themselves revealing of the dynamics of scientific cooperation with the southern shore of the Mediterranean. The ENI CBC “Mediterranean Sea Basin Programme” (2014–2020), under the European Neighbourhood Instrument, was among the first to promote cross-border collaboration between EU Member States and southern Mediterranean neighbours, addressing shared challenges such as the environment and cultural heritage. By contrast, Interreg Euro-MED (2000–2027) has remained largely inward-looking, supporting cooperation only among EU Mediterranean regions, although it has included a thematic pillar specifically dedicated to the protection and valorisation of natural and cultural heritage. Its successor, Interreg NEXT MED (2021–2027), marks a significant shift by extending funding to southern and eastern Mediterranean partners, thereby strengthening their direct access to EU resources. Finally, Creative Europe (since 2014) formally allows the participation of certain non-EU countries, such as Tunisia, though its scope remains predominantly Europe-centred.

Taken together, these programmes illustrate a gradual yet uneven trajectory: while ENI CBC Med and Interreg NEXT MED offer more inclusive frameworks, Euro-MED and Creative Europe still reveal persistent Eurocentrism and limited structural participation for southern partners. At the same time, the current Horizon Europe Work Programme includes calls for proposals that explicitly target cooperation with the Mediterranean region, signalling a potential step towards more balanced engagement.

In December 2024, the European Union established for the first time the position of Commissioner for the Mediterranean. This institutional innovation reflects the EU’s renewed strategic focus on the Mediterranean region, aiming to strengthen political engagement, improve cross-policy coordination, and address key challenges such as migration, regional stability, energy security, and climate change.

Although the mission letter from Commission President Ursula von der Leyen to Commissioner Dubravka Šuica does not explicitly mention cultural heritage, the Commissioner, in a speech delivered in February 2025, emphasised her intention to integrate a strong cultural dimension into the forthcoming New Pact for the Mediterranean. This includes the protection of cultural heritage, the promotion of tourism, cooperation between museums, and academic exchange programmes.

Additionally, the New Pact for the Mediterranean, which is currently under discussion, is expected to be presented in the autumn of 2025. In preparation, regional consultations are being held to help define its policy priorities, and the European Institute of the Mediterranean (IEMed) has launched an open regional consultation, the Euromed Survey 2025, to gather input from policymakers, experts, civil society actors, and private sector stakeholders ( IEMed & EuroMeSCo, 2025).

We may therefore conclude that these initiatives reflect the EU’s commitment to deepening relations with Southern Mediterranean countries and to jointly addressing shared challenges.

### The Role of the UfM – Union for the Mediterranean

**Figure 1.**
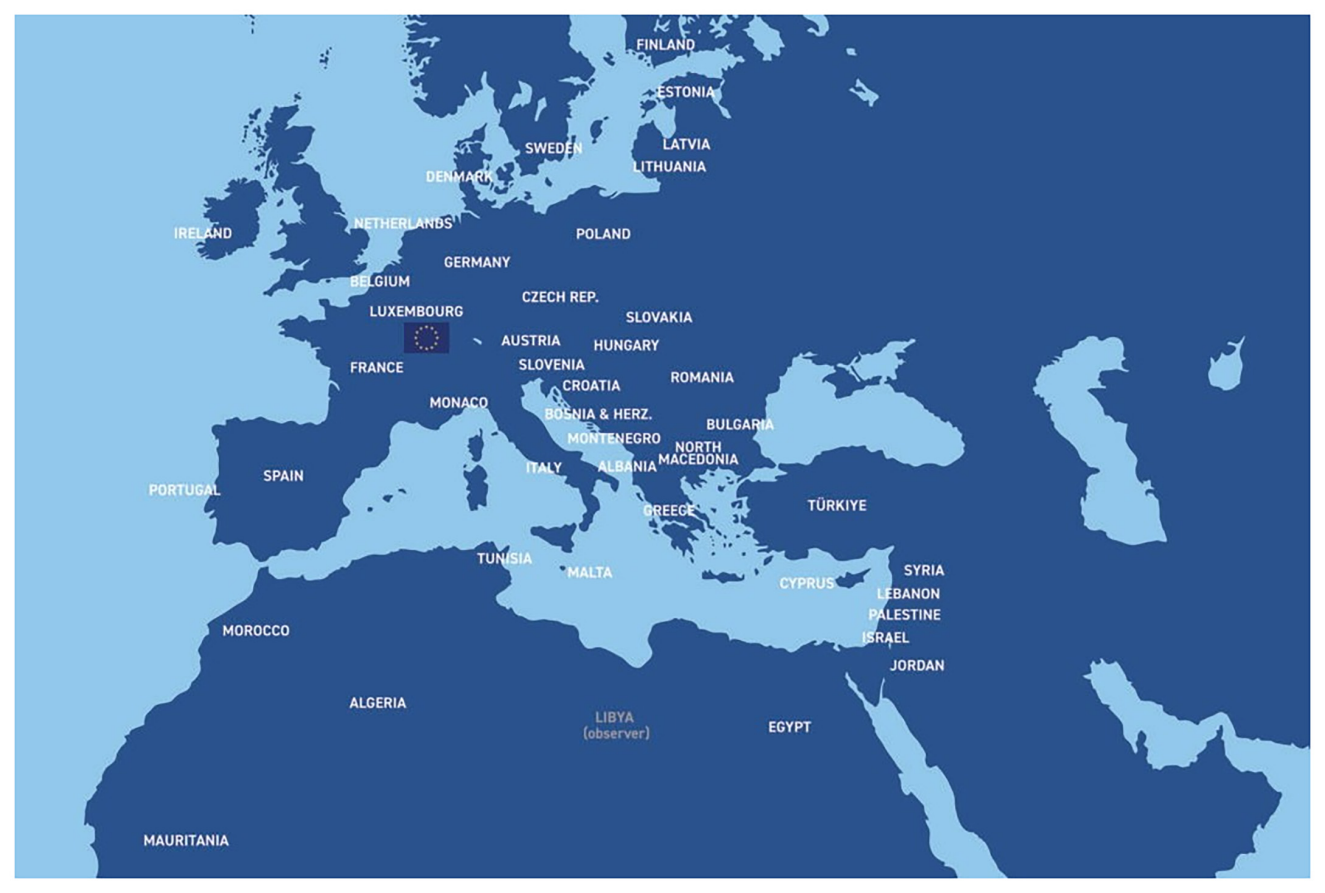
Union for the Mediterranean (UfM) members, comprising the 27 European Union member states and 16 countries from the southern and eastern Mediterranean shores (Map source: Union for the Mediterranean).

The Union for the Mediterranean (UfM), launched in July 2008, is an intergovernmental Euro-Mediterranean organisation that brings together all 27 European Union member states and 16 countries from the southern and eastern shores of the Mediterranean ( Figure 1). The UfM Secretariat is based in Barcelona, as the initiative is a direct continuation of the Barcelona Process and the Barcelona declaration ( UfM, 1995). As the successor to the Euro-Mediterranean Partnership, the UfM’s main objective is “to strengthen regional cooperation, dialogue, and integration in the Euro-Mediterranean area”, thereby contributing to the creation of an “area of peace, security, stability, and shared prosperity” ( https://ufmsecretariat.org/).

The bibliography that specifically addresses the role of the UfM in the promotion and enhancement of cultural heritage remains limited, although some authors have offered valuable insights. The literature indicates that, while the UfM has made progress in communication and in engaging civil society, it continues to face significant obstacles, including political instability and the under-representation of certain stakeholders. At the same time, cultural heritage is recognised as a unifying force that fosters a shared Mediterranean identity and facilitates dialogue. UfM initiatives in this area have centred on the preservation of historical sites and cultural practices, thereby contributing to sustainable tourism and economic development. Nevertheless, cooperation on cultural heritage remains constrained by limited funding, ongoing political instability, and a lack of sustained commitment from some member states ( Elistania *et al.*, 2019).

While culture, heritage, and the creative industries are not listed among the UfM’s six main sectors of cooperation – three under Inclusive Development (Economic Development & Employment; Higher Education & Research; Social & Civil Affairs) and three under Sustainable Development (Transport & Urban Development; Water, Environment & Blue Economy; Energy & Climate Action) – an in-depth review of the UfM’s work programmes and reports reveals that cultural heritage has nevertheless emerged as a cross-cutting theme in several initiatives. Despite the absence of a formal cultural sector, the UfM’s actions reflect a growing awareness of the strategic value of cultural heritage as a driver of identity, sustainability, and resilience. 

Through a careful analysis of the reports and work programmes available on the UfM website ( https://ufmsecretariat.org/), covering documents produced between 2015 and 2025 to span a decade of action, it was possible to identify four thematic areas of the UfM’s concrete involvement in the field of cultural cooperation, including cultural heritage. They include:

a) Heritage Protection: preservation and rehabilitation of heritage, both in the face of natural and human disasters.
b) Fostering a Common Euro-Mediterranean Identity based on culture, historical, civilizational, and linguistic heritage: the creation of the Euro-Mediterranean University of Fes, the “Mediterranean Day” initiative, and the “Mediterranean Capitals of Culture and Dialogue.”
c) Urban Sustainability and Heritage: Focused efforts on the regeneration of historical cities within the broader UfM GreenerMed agenda.
d) Cultural and Sustainable Tourism: Emphasis on the role of culture in regional attractiveness, sustainability, and innovation.

Starting in 2015, the “Regional Platform for the Development of Culture and Creative Industries and Clusters in the Southern Mediterranean Countries” was mentioned in several reports in the following years ( UfM, 2016; UfM, 2018). The identification of 144 clusters across seven countries demonstrated the potential for developing this sector. Workshops and training sessions were implemented to improve quality and explore collaborative markets in both Europe and the Gulf. However, the project remained more focused on artistic sectors than heritage itself. In 2018, the initiative was extended for another 18 months due to the success of its first phase ( UfM, 2019). That same year also marked the beginning of UfM activities in the field of tourism, particularly centered on sustainability through SME digitalization and strategies for energy efficiency and climate action.

Urban development provided another key entry point for cultural heritage. In 2016 and 2018, reports highlight a joint initiative between the UfM and financial institutions such as the European Investment Bank to support sustainable urban planning across Mediterranean cities ( UfM, 2018). Among these efforts, the creation of an “eco-city” in Morocco’s Bouregreg Valley, in collaboration with the Bouregreg Valley Development Agency (AAVB), included the preservation and rehabilitation of cultural heritage – specifically the restoration of a necropolis designated as a UNESCO World Heritage Site ( UfM, 2019).

In 2019, the report highlighted the first graduation ceremony of “young Euro-Mediterranean leaders” from the Euro-Mediterranean University of Fes (UEMF) ( UfM, 2020). Endorsed by the 43 Member States of the UfM in 2012, the Euro-Mediterranean University of Fes (UEMF) is one of the UfM’s flagship projects and forms part of the Mediterranean Initiative for Jobs (Med4Jobs). Aligned with the conclusions of the UfM Ministerial Conference on Strengthening Cooperation through Research and Innovation (Valletta, May 2017), the 1st Euro-Mediterranean Ministerial Conference on Higher Education and Scientific Research (Cairo, 2007), and the recommendations of the European Neighbourhood Policy (2015), the promotion of international student mobility stands as a key priority for the University. This university seeks to foster intercultural dialogue and cooperation through a curriculum that spans engineering and the humanities, while incorporating transversal topics such as history, civilization, heritage, and the languages of the Euro-Mediterranean region.

In the 2020 report, the launch of the “Day of the Mediterranean”, officially celebrated on 28 November, marked a turning point in the promotion of a common Mediterranean identity ( UfM, 2021). The celebration encourages cultural exchanges and public participation by highlighting the richness of Mediterranean traditions, heritage, and shared values. Activities have included the symbolic illumination of historic buildings with the “Day of the Mediterranean” logo and the free or special opening of heritage sites across the region. Cities like Barcelona, Cairo, and Alexandria have taken part in this growing initiative. The same year also saw the UfM respond to the devastating explosion in Beirut’s port area. Three strategic meetings were held to assess damage and develop a plan for the “Recovery and Reconstruction of the Built Heritage and Historic/Regular Residential Buildings”, emphasizing heritage as an essential part of Mediterranean identity.

The following year, 2021, they reinforced the urgency of the green transition, with a particular emphasis on natural heritage. A key event was the workshop “Living with World Heritage: Adaptive Reuse and Regeneration for Sustainable Cities”, organized in partnership with the UNESCO World Heritage Center. It brought urban regeneration and sustainability – especially in historical cities – into the spotlight ( UfM, 2022a). The first full celebration of the “Day of the Mediterranean” also took place that year, featuring strong engagement from civil society, the selection of a logo translated into 13 languages, 21.000 website visits, and 4.1 million video views on the campaign “In a single word – What does the Mediterranean mean for you?”. The event also hosted the “6th UfM Regional Forum” in Barcelona with over 150 “high-level participants”.

In the 2022 report, concern for cultural heritage protection in the context of disasters became even more prominent. The PROCULTHER project (“Protecting Cultural Heritage from the Consequences of Disasters”) was highlighted as a notable initiative ( UfM, 2023a). Countries like Italy, France, Spain, and Turkey, with recognized experience in cultural emergency response, were joined by Germany and Portugal to develop coordinated strategies. Media impact and social media engagement related to the “Day of the Mediterranean” were also emphasized in the year’s report.

Even in 2022, the first Conference of the Ministers of Culture of the Euro Mediterranean Region took place in Naples on 16–17 June, culminating in the Naples Declaration, which aims at enhanced cooperation and joint action. Among its key outcomes was the proposal to establish a "Mediterranean Capital of Culture", inspired by the success of the European Capital of Culture initiative and intended to promote intercultural dialogue and shared heritage across the Mediterranean. Europa Nostra participated as a special guest in discussions concerning strategies for the protection and enhancement of the Euro Mediterranean region's cultural heritage, underlining the vital role of civil society in safeguarding cultural values and fostering regional cohesion ( Europa Nostra, 2025).

By 2023, cultural heritage was further addressed through the lens of climate and environmental challenges, in line with the “UfM 2030 GreenerMed agenda”. Particular attention was given to the issue of land abandonment and its consequences for ecology, agriculture, and cultural landscapes ( UfM, 2024a). In October, the “Young Leadership Programme Mediterranean 2023”, organized in partnership with the European Forest Institute, brought together young participants from Algeria, Egypt, Morocco, Tunisia, and Turkey in Barcelona to explore innovative solutions. The focus was on scientific knowledge, social strategy, youth leadership, and sustainable entrepreneurship related to biodiversity and heritage preservation.

Also in 2023, the UfM launched the “Mediterranean Capitals of Culture and Dialogue”, an initiative that emphasizes cultural diversity and shared identity. Developed in cooperation with civil society and member states, this initiative selects two cities each year – one from the north and one from the south – to serve as cultural ambassadors culminating their programmes on 28th November. The objective of the initiative is to unite the peoples of the Mediterranean by promoting a common identity through three key actions: fostering Euro-Mediterranean identity, encouraging territorial cooperation between cities, and adopting a bottom-up approach that strengthens collaboration between local governments and civil society. By the end of 2023, Alexandria (Egypt) and Tirana (Albania) were named the first “Mediterranean Capitals of Culture and Dialogue”.

Finally, in the “2024 Work Programme”, two workshops from the “Traditional Building Skills in the South Mediterranean” program were highlighted ( UfM, 2023d). This program aims to integrate tourism planning and heritage management, developing sustainable tourism and creating job opportunities. The continued support for the “ECWMF-Copernicus” program in the development of apps like the “Energy App” for disseminating information on the impact of sea-level rise on cultural heritage sites was also noted.

Having reviewed the reports and work programmes, we now turn our attention to two UfM initiatives that warrant specific consideration – the “Day of the Mediterranean” and the “Mediterranean Capitals of Culture and Dialogue”.

The “Day of the Mediterranean” was born in November 2020. All Member States of the Union for the Mediterranean declared the 28th of November as the official Day of the Mediterranean, to be marked annually, calling upon the entire Mediterranean community and everyone who identifies with the Mediterranean to celebrate its legacy ( UfM, 2024b). The “world-class cultural heritage sites”, along with biodiversity and geodiversity, traditions, history, philosophy and education, among other relevant cultural legacies, justify this one-day annual celebration. It aims at encouraging the organisation of events, the launch of initiatives as well as leverage media attention, fostering a common and shared Mediterranean identity. Each year, they launch a new communication and social media campaign. The main objective is to understand what unites the different peoples of the Mediterranean, what do they share and how can it be used for their future as an identity area.

In the reports available on the “Day of the Mediterranean” website ( https://mediterraneanday.com/), it was possible to reflect on the key actions and some of the themes that drove the various campaigns and activities launched by this celebration.

At the first celebration, in 2021, they highlighted the website translated in 5 languages, the engagement with civil society and a collaborative agenda where stakeholders and institutions published their activities related to the Day of the Mediterranean: 75+ activities in 18 countries from the region ( UfM, 2022b). A wide array of thematics were covered: climate change, energy, environmental safeguarding, water, food security and agriculture, gender equality, entrepreneurship, intercultural and interreligious dialogue, trade and investment, youth, urban development and transport, science and technology, migration, architecture, culture, art and foreign policy – among many others. Also, to engage with citizens, they launched the “Mediterranean in 1 word” and the “What is the Mediterranean” video campaigns.

In the celebration of 2022, we continued to see a commitment to publicising the “Day of the Mediterranean” in the media and on social networks. This year’s edition invested on the “senses” and the idea of “mediterraneanity” ( UfM, 2023b). They launched the campaigns “The Mediterranean, a journey through the senses” and “If you think of the Mediterranean, where does it take you?”.

For the 2023 edition, no full report is available for download. However, the highlights presented on the UfM website provide an overview of the campaign, which proposed to “Find the Mediterranean in your city” ( UfM, 2023c).

In 2024, the campaign almost seemed like a theme: “The Next Wave: Building a shared tomorrow”. The activities shown in the report include music concerts, young influential digital content creators, a lottery ticket with the Day of the Mediterranean’s visual identity, non-governmental and cultural organisations to raise awareness on challenges and innovative solutions, high school students reflecting on the biodiversity and environmental sustainability of the Mediterranean, as well as the importance of preserving natural heritage, etc ( UfM, 2025).

The Day of the Mediterranean thus seems to be establishing itself as a wide-ranging celebration of Mediterranean diversity in all its different dimensions, being possible to incorporate cultural heritage in many ways, giving room for individual, personal or institutional initiative.

The other initiative launched by the UfM that we highlight is the “Mediterranean Capitals of Culture and Dialogue”. To evaluate the activities carried out in this context, the programmes of the capitals selected for 2025 and 2026 were analysed.

The first selected capitals were Alexandria (Egypt) and Tirana (Albania), for 2025. Tirana was chosen for its unique convergence of cultures, religions, and historical influences. Its programme, titled “MediTIRANEan Bridges”, is based on four pillars: cultural exploration, artistic creativity, digital heritage, and sustainable tourism. Activities included the celebration of the International Day of Sites and Monuments on 18 April, the International Festival of Aquarelle in May, and festivals such as “Vere N’Shesh” and the “Olive Fest”, held in historic sites like the Castle of Petrela, the Illyrian city of Persqopi, and the Lake of Gurres. Alexandria was selected for its intellectual and cultural traditions, as well as its dedication to intercultural dialogue and the values of tolerance. Its programme focused on cultural heritage through events such as “Risk to World Heritage Sites across the Mediterranean from rising sea levels”, addressing the vulnerabilities of 49 coastal UNESCO sites, including Alexandria, Carthage, Venice, and Delos.

For 2026, the chosen capitals are Tetouan (Morocco) and Matera (Italy). Tetouan is celebrated for its Andalusian legacy and multicultural past, rooted in Phoenician, Roman, and Islamic traditions. While the full activity programme is yet to be announced, Tetouan aims to partner with Matera in organizing joint exhibitions and artistic collaborations. Matera, previously European Capital of Culture in 2019, proposed a new programme titled “Terre Immerse”, highlighting the Mediterranean’s deep historical and cultural ties. It includes festivals, film screenings, and live performances, and places strong emphasis on community involvement and the amplification of local voices.

The cultural programmes of the “Mediterranean Capitals of Culture and Dialogue” reinforce patterns already observed in the UfM’s broader initiatives, notably a clear concern for the protection of cultural heritage – particularly in response to natural and human-induced disasters.

This analysis shows a recurring effort to build a shared Euro-Mediterranean identity based on historical, civilizational, and linguistic heritage. Sustainability is pursued through the integration of heritage into urban development, especially under the “GreenerMed agenda”, while tourism is strategically linked to sustainability and digital innovation. The Mediterranean, therefore, emerges not only as a geopolitical space but as a vibrant cultural ecosystem, capable of fostering a renewed, resilient, and plural Euro-Mediterranean identity. Over the past decade, UfM’s actions consistently engage cultural heritage as a platform for cooperation, resilience, and identity-building, focusing on shared values, historical awareness, sustainable development, and the unifying power of culture in times of crisis. The Mediterranean represents both strategic geopolitical importance and a cultural force for a cohesive European identity rooted in its southern legacies.

### European cultural heritage policies and initiatives: Mediterranean contributions to a shared European identity

European cultural heritage plays a crucial role in shaping the narratives of EU identity and represents an expanding, yet contested, area of policymaking within the European Union. The formal recognition of a shared cultural legacy was first expressed in the 1973 Declaration on European Identity. Since then, EU heritage policies have evolved significantly, both in scope and in number.

Among the most notable recent initiatives is the European Heritage Label (EHL), established in 2011 by a decision of the European Parliament and the Council of the European Union (Decision No. 1194/2011/EU). This initiative exists alongside an older and broader programme that extends beyond the borders of the European Union – the Cultural Routes of the Council of Europe.

Recent research shows that, over the past few decades, the EU has increasingly employed cultural heritage as a strategic tool to promote cohesion among its member states and to strengthen relationships with non-EU countries. Heritage initiatives have sought to foster a shared sense of European identity and to support integration, while more recently also addressing global crises by leveraging culture to build dialogue and cooperation both within and beyond EU borders ( Lähdesmäki *et al.*, 2020; Mäkinen *et al*., 2023).

Considering this, we find it particularly relevant to explore Mediterranean cultural heritage within the framework of the EHL, as well as to examine it in the context of the Cultural Routes of the Council of Europe. It is to this subject that we now turn in the following pages.

***The European Heritage Label (EHL).*** The European Heritage Label (EHL) became an official EU initiative in 2013, coordinated by the European Commission ( https://culture.ec.europa.eu/cultural-heritage/initiatives-and-success-stories/european-heritage-label). Since then, it has been awarded every two years to recognize the outstanding European value of cultural heritage sites and to reward their efforts in promoting the European project.

Created in 2023, the EHL Bureau manages the EHL community and supports its development. It works to foster synergies among EHL sites, strengthen capacities, and enhance their visibility.

At the moment, the EHL includes over 67 sites across Europe. It aims to bring the European narrative closer to citizens by highlighting heritage that is meaningful to communities and contributes to the construction of Europe.

Some authors have analysed the importance of the EHL within the context of the European Union’s cultural policy and identity-building efforts ( Calligaro, 2014; Cabbar, 2019; Mäkinen *et al*., 2023). These studies highlight how the EU leverages initiatives like the European Heritage Label to promote cultural diversity and shared values, reflecting a more inclusive understanding of European identity. Furthermore, the EHL is seen as a diplomatic instrument to strengthen international cultural relations, support peace and stability, and communicate a cohesive European narrative both within and beyond its borders.

As part of our analysis, we aim to understand how the EHL sites connected with the space, history, and memory of the Mediterranean intertwine the discourse of Mediterranean legacy with that of European significance.

To this end, we draw on information provided by the European Commission website regarding site eligibility criteria and the selection process, as well as the “Control procedures” and the “Withdrawal or renunciation of the European Heritage Label” ( European Commission: Directorate-General for Education, Youth, Sport and Culture, 2022).

Among the 67 sites awarded the European Heritage Label (as of 2023), several can be clearly identified as having a distinctly Mediterranean character or as being readily associated – albeit at times indirectly – with Mediterranean cultural heritage. Notable examples include: the Heart of Ancient Athens (Greece), Ostia Antica (Italy), the Archive of the Crown of Aragon (Spain), and the Archaeological Site of Nemea (Greece).

A careful examination of the information available on the official EHL website, alongside with the Evaluation Reports, the Monitoring Reports and the European Panel Reports, supports the conclusion that the Mediterranean functions as a geographical, historical, and cultural frame of reference for European identity within the context of EHL sites.

However, these sites tend – perhaps deliberately – to avoid direct references to the term “Mediterranean” in their core narratives. Instead, they embed it as an underlying historical and cultural structure within their articulation of European significance. In effect, the narratives adopted reinforce the Mediterranean as a foundational identity matrix for Europe, even when it is not explicitly named.

To assess how the Mediterranean dimension is reflected in the European Heritage Label initiative, we examined the 67 designated sites. Particular attention was given to those that either explicitly reference the Mediterranean or can be indirectly associated with its cultural, historical or geopolitical legacy.

Table 1 presents a selection of these seven sites highlighting their Mediterranean character, the core European values they express (or may express), and the documentary sources supporting these interpretations. This comparative synthesis illustrates how, through the lens of the EHL, *the Mediterranean has its say* in shaping a shared European identity.

**Table 1.** Mediterranean EHL Sites and European Values.

EHL SITE	COUNTRY	MEDITERRANEAN CHARACTER	QUOTES	RELEVANT EUROPEAN VALUES	SOURCE / REPORT
**Sagres Promontory**	**Portugal**	Maritime gateway linking Atlantic and Mediterranean	“Sagres Promontory became the privileged scenario for the accomplishments of the ‘Age of Discoveries’ in the fifteenth century, a key historical moment that marked the expansion of European commerce, science, and technology both towards the Atlantic and the Mediterranean, setting Europe on its path to the global projection that has come to define the modern world.”	Scientific research and development, education and innovation, mobility, european global projection, intercultural dialogue	*Monitoring Report 2020*, 33 ( European Comission, 2020)
**Heart of Ancient Athens**	**Greece**	Classical heritage rooted in the Mediterranean	“The Heart of Ancient Athens comprises a historical landscape where events which helped shape some of the most essential aspects of European identity took place, from the development of classical art and theatre to democracy, philosophy, logic, equal rights and sciences.” “Athens, the leading cultural centre of ancient Greece, [is] the cradle of essential aspects and values of European culture and civilisation.” “It shaped or influenced all kinds of art from antiquity up to the present time.”	Democracy, philosophy, equality, rule of law, respect for human rights, active citizenship, freedom of expression and thought, arts	EHL Website *Monitoring Report 2020*, 15 ( European Comission, 2020)
**Ostia Antica**	**Italy**	‘Gateway to the Mediterranean’, Roman trade hub	“The Archaeological Area of Ostia Antica is a place where goods circulated, and different cultures and religions mingled. As a gateway to Rome Ostia was a melting pot of the different people who lived under the Roman Empire and a place with far-reaching influence on land, across the Mediterranean basin and beyond. Evidence of the trade, the exchanges and the diverse population is still visible today in the mosaic floors, the archaeological remains and funeral inscriptions.” “Ostia Antica, promoted as a gateway to the Mediterranean and a melting pot of cultures, beliefs, and goods from around the world, serves as an excellent starting point for discussions within the diverse, multi-ethnic community of modern-day Ostia.”	Intercultural exchange, mobility, transregional connectivity	EHL website *Monitoring Report 2024*, 27 ( European Comission, 2025)
**Archive of the Crown of Aragon**	**Spain**	Mediterranean political and administrative legacy from the Middle Ages and Early modern period	“It serves as a centralised depositary for the Crown of Aragon, a monarchy that extended across the Mediterranean”	Historical memory, documentary heritage, rule of law, cultural diversity	*Monitoring Report 2020*, 29 ( European Comission, 2020)
**Archaeological Park Carnuntum**	**Austria**	Roman trade routes connecting the Danube to the Adriatic and the Mediterranean world	“Carnuntum witnessed important events and, being situated at the border between the eastern and western halves of the Roman Empire, was of economic and strategic importance and a multicultural place.” “The European significance of the site, centered around the Roman route network and the region’s multicultural heritage, is a key theme in its narrative.”	Multicultural integration, transregional connectivity, multicultural heritage, cultural diversity	*Monitoring Report 2024*, 31 ( European Comission, 2025)
**Archaeological Site of Nemea**	**Greece**	Panhellenic Mediterranean site linked to Hercules and ancient mythology	“The site includes the sport roots of Europe and the ideals of classical sports as an element for the comprehensive education of young people.” “The Games are connected to the site’s European importance, emphasizing the historical role of sports in youth education and fostering peaceful competition.”	Peaceful competition, youth education, equality, social inclusion, tolerance	EHL Website *Monitoring Report 2024*, 23 ( European Comission, 2025)
**Thracian Art in Eastern Rhodopes: Aleksandrovo Tomb**	**Bulgaria**	Link between Thracian culture and the classical Mediterranean world.	“The Museum Centre showcases the ancient Thracian civilisation, one of the earliest cultures in Europe. It promotes understanding of this culture’s influence on subsequent European civilizations.”	Intercultural influence, intercultural dialogue, multicultural heritage	*Monitoring Report 2024*, 25 ( European Comission, 2025)

The analysis of EHL sites with a Mediterranean character clearly demonstrates that the Mediterranean serves as a geographical, historical, and cultural frame of reference for European identity. These sites, spread across various Mediterranean and para-Mediterranean regions, embody multiple dimensions of the European Union’s foundational values, reflecting both civilisational continuities and historical transformations that gave rise to new political and cultural visions.

However, some of these sites – whether in the information provided on the EHL map or on their official websites – do not always articulate clear connections with the Mediterranean context, even though for others, such as Nemea and Athens, the link is self-evident.

Classical Antiquity, represented by sites such as the *Heart of Ancient Athens*, *Nemea* and *Ostia Antica* reveal how values such as democracy, equality, active citizenship, cultural diversity, and intercultural exchange are deeply rooted in Mediterranean experiences of coexistence, trade, and political thought. Athens stands as a symbol of democracy and philosophy; Ostia embodies the plurality and cultural dynamism of the Roman Empire; and Nemea reflects the role of sports in youth formation and peaceful coexistence – echoing values of education, equality, and social inclusion.

The *Archive of the Crown of Aragon* holds documents of great importance for the history of Europe. It reflects how key European values – such as historical memory, the rule of law, cultural diversity, and multilingual governance – were already present in the medieval Mediterranean. As the central archive of a transregional monarchy, it preserves a legacy of legal pluralism, administrative cohesion, and coexistence among diverse communities, anticipating modern European principles of justice, unity in diversity, and institutional accountability.

The *Sagres Promontory*, at the western edge of the Mediterranean basin, demonstrates Portugal’s role as a bridge between the Mediterranean and the Atlantic, integrating scientific and technological expansion into the broader context of the Age of Discoveries – anticipating modern ideals of mobility, global projection, and solidarity.

Sites such as *Carnuntum* and the *Aleksandrovo Tomb*, although geographically beyond the core Mediterranean region, show profound cultural interconnections with the classical Mediterranean world, revealing how the Greco-Roman legacy shaped inland Europe through trade routes, cultural exchange, and artistic and religious influence – reinforcing values of multiculturalism, social cohesion, and unity in diversity.

These sites demonstrate how this space has been – and remains – essential to the shaping of a shared European identity. Each site, in its own way, embodies values such as human dignity, democracy, social justice, tolerance, and historical memory, and reveals how the Mediterranean is more than a sea – it is a structuring idea of Europe.

The role that Mediterranean cultural heritage may play in shaping what is – or could be – considered “European heritage” gains increasing relevance considering the findings of the 2024 EHL Monitoring Report ( European Commission: Directorate-General for Education, Youth, Sport and Culture, 2025). The report stresses the urgent need for deeper reflection on how European significance is assessed, communicated, and experienced by communities. It calls for a renewed examination of the relationship between site projects and their European relevance, proposing that this issue be addressed in the forthcoming revision of the EHL’s legal basis. It also emphasises the importance of enhancing the visibility and public understanding of European significance at the sites themselves.

This recommendation is extended to sites with strong associations to Mediterranean heritage (such as those analysed in this text), with a particular emphasis on strengthening the discourse around shared European values.

Notably, the same Monitoring Report underlines the potential of transborder regional cooperation as a structuring principle for the EHL, highlighting historical-geographical areas such as the Danube Valley, the Polish Lithuanian Commonwealth, and particularly the Mediterranean. While it also proposes thematic subgroups (e.g. art, community values, heritage institutions, landscape and environment, music), the regional dimension emerges as a key framework for fostering connections between sites and could provide a more grounded basis for articulating European significance.

The analysis of previous evaluation and selection reports also offers some positive insights and recommendations for the Mediterranean-linked sites discussed in this study.

The 2018 evaluation report provided the first comprehensive overview of activities developed by the selected sites ( European Commission: Directorate-General for Education, Youth, Sport and Culture; PPMI & EDUCULT, 2019). Several were praised for their innovative and engaging approaches. The *Archaeological Park Carnuntum*, in Austria, was commended for its immersive reconstructions and use of modern technology to bring Roman history to life. Similarly, the *Archive of the Crown of Aragon*, in Spain, was highlighted for its exemplary management and successful digitalisation efforts, which made its extensive collections accessible to a broader public. The *Thracian Art in Eastern Rhodopes: Aleksandrovo Tomb*, in Bulgaria, was also recognised for effective preservation and for promoting the unique European significance of this archaeological site.

However, the report also identified recurring challenges, particularly in the communication of European relevance and increasing visibility at the European level. The *Sagres Promontory*, in Portugal, despite its strong historical value related to the Age of Discoveries, was seen as lacking a cohesive narrative and engaging communication strategies. The *Archaeological Site of Nemea*, in Greece, faced similar issues, with the report recommending that it better promote its connection to the pan-Hellenic games and its broader European importance to attract a more diverse audience.

As we can observe, the 2018 Evaluation Report offers no indication that the Mediterranean character of these sites was considered a contributing factor in reinforcing their European significance. This notion appears to emerge only at a later stage, particularly in the discourse of the 2024 EHL Monitoring Report, which introduces the potential of transborder regional cooperation as a structuring principle for the Label. By highlighting historically and geographically defined areas – such as the Mediterranean – the report opens the door to a more place-based and interconnected understanding of European significance. However, it remains unclear how sites might effectively engage with this dimension, and, more importantly, how they can avoid falling into a static, history-bound narrative while instead aligning their discourse with contemporary European society.

The concept of “European identity” is, of course, highly debatable, and several authors have examined how it is approached within the EHL framework ( Čeginskas & Kaasik-Krogerus, 2022; Kaasik-Krogerus, 2020). These scholars argue that the EHL often falls short in its attempt to meaningfully connect institutional actors and the wider public to a sense of European identity. They highlight the complexity and limitations of EU identity politics as expressed through heritage policy, noting the vague and instrumentalised use of the notion of “dialogue” in EHL narratives. While official documents present dialogue as a positive European value associated with peace, diversity, and cooperation, the term remains poorly defined and functions largely as a floating signifier, adapting to different contexts without clear explanation of who participates, how, and under what conditions. In practice, dialogue appears explicitly in fewer than 15% of EHL site reports, and even then, it is often invoked symbolically in relation to the past rather than as a genuine, ongoing process of negotiation in the present ( Čeginskas & Kaasik-Krogerus, 2022).

Focusing on our seven case studies, we can conclude that they are not just physical landmarks, but guardians of a vast legacy that encompasses various dimensions of European identity. Some of the selected sites reach back to the continent’s roots, highlighting their historical and archaeological origins. They offer a glimpse into the ancient civilisations, cities, and cultures that shaped Europe, revealing the foundations on which modern societies were built. Other sites represent the richness of European intellectual culture, encompassing higher education institutions, science centres, and spaces where great ideas flourished. They bear witness to Europe’s commitment to knowledge, innovation, and progress. These are sites that contribute to the cultural foundations of European identity and to the history of Europe, full of intersections, but whose European significance is not directly associated with the history of the European Union itself, such as *Carnuntum* or the *Aleksandrovo Tomb*.

***The Cultural Routes of the Council of Europe.*** The Cultural Routes of the Council of Europe (CRCE) were established by the Council of Europe in 1987. This initiative crosses diverse European cultures and regions that have collectively contributed to heritage, thus combining space and time. They thus articulate transnational historical narratives, focusing on shared values (such as human rights, democracy, diversity).

According to the official website ( https://www.coe.int/en/web/cultural-routes/overview), the Council of Europe Committee of Ministers Resolution (CM/Res(2013)66) confirming the establishment of the Enlarged Partial Agreement on Cultural Routes (EPA), defines a Cultural Route of the Council of Europe as: “ *a cultural, educational heritage and tourism co-operation project aiming at the development and promotion of an itinerary or a series of itineraries based on a historic route, a cultural concept, figure or phenomenon with a transnational importance and significance for the understanding and respect of common European values.*”

The effort to build a European identity is, once again, clearly reflected in this initiative of the Council of Europe. However, this analysis seeks to go further by reflecting on the role of the Mediterranean in shaping these routes. How is the Mediterranean mobilised within the discourse of “European identity”? To explore this, we analysed the activities of ten selected routes in the region and examined related documentation, including the certification criteria, the certification cycles, and the evaluation reports of the applications for certification, available on the official website ( https://www.coe.int/en/web/cultural-routes/certification).

These routes have been selected to examine how Mediterranean culture is represented (or not) within the framework of the Cultural Routes programme. The following provides a brief description of each route, along with relevant information.

**Phoenicians’ Route**

Due to its characteristics, this seems to us to be one of the main routes in the Mediterranean. Certified in 2003, it includes countries such as Lebanon, Italy, Spain, Tunisia, Greece, etc., and follows the ancient sea and land routes used by the Phoenicians, connecting various archaeological and cultural sites along the Mediterranean. This region is highlighted as a space for intercultural exchange and dialogue, reinforcing the idea of a common European heritage based on diversity and cultural exchanges, as a path to peace: “ *This network is a way to work together for the development of peace and mutual respect in the Mediterranean.*” ( https://www.coe.int/en/web/cultural-routes/the-phoenicians-route). However, although this route sees the number of its partners increasing from year to year, the network faces difficulties on the southern shore of the Mediterranean, a key region for the construction of the main objective of the route – intercultural dialogue ( Guérin, 2021–2022). On the other hand, the activities carried out within the framework of this route have mobilised countries such as Spain and Italy, which have organised meetings entitled “Euro-Mediterranean Dialogues”, between researchers studying the battle of Baecula (Spain) and the battle of Metaurus (Italy), as well as exhibitions on Hannibal's expedition, in Capua, Cretone (Italy). On the other bank, and despite the difficulties indicated above, dialogue partnerships have been established between members of this route and public institutions in Jordan, Palestine and Lebanon. The network of this route also includes schools in the Mediterranean, organizing events to raise awareness of the Mediterranean cultural heritage. The initiatives of this route have grown over the years, and it is also foreseen the possibility of extending the network beyond the Mediterranean, thus covering universities dedicated to Phoenician studies, such as the University of Leuven and the University of Oxford.

**Routes of** *El legado andalusí*

Certified in 1997, the route crosses Spain, Morocco, Tunisia, among others. It focuses on the Islamic heritage in the Iberian Peninsula, especially Andalusia, and its connections with North Africa, the eastern Mediterranean and Latin America ( https://www.coe.int/en/web/cultural-routes/the-routes-of-el-legado-andalusi). Spain and the region of Andalusia stand out as “cultural bridges” between East and West. The 2023–24 report also highlights the richness of the “cultural mix” that is the Andalusian legacy: it mixes elements from the Mediterranean, the Middle East and Europe, giving rise to new forms of artistic and scientific expression, as well as a richer dialogue in the cultural and social framework ( Moreno-Martín, 2023–2024). This itinerary thus promotes interaction along the Mediterranean, whether in educational or tourist terms. However, most of the activities take place in Spain, although in 2023 the event “Euro-Mediterranean Cultural Dialogue: Celebration of the Andalusian Days in Lebanon” was held in Beirut. In this regard, the same report mentions the priority need to improve the dialogue between Spain and the other members of the route, since there are no joint action strategy or regular meetings between these parties. The same problems had already been pointed out previously, in the 2019–20 report ( Brianso, 2019–2020). Even so, the work network of this route has expanded, working together with other markedly Mediterranean routes, such as the Aeneas Route, the Routes of the Olive Tree, the Iter Vitis Route and the Phoenicians Route.

**Routes of the Olive Tree**

Greece, Italy, Spain, Tunisia, Morocco, among others, appeared as part of this route focused on the cultivation of the olive tree as a symbol of Mediterranean civilization (“the olive tree civilization”) ( https://www.coe.int/en/web/cultural-routes/the-routes-of-the-olive-tree). The importance of olive oil cultivation over the millennia is reflected in trade, food and the commercial and cultural bridges created over time between the various regions of the Mediterranean. This route celebrates the olive tree as a common element of the Mediterranean, which unites European and Mediterranean countries and reflects a common diet. The 2019–2020 evaluation report misses the importance of the olive tree for European culture: “ *the main tasks of the Foundation highlights a profoundly European heritage, the olive, and the values symbolized by the tree: a heritage both tangible and intangible, with a message of peace and intercultural dialogue; the values of the Mediterranean landscape; human values embodied by the work of the Mediterranean populations who got from this tree, since antiquity, their livelihoods.*” ( Gravari-Barbas, 2019–2020) However, the same document also points out some problems, in terms of management and implementation of actions. The route network has been decreasing since 2018, which is partly explained by the Arab crisis, which has affected the southern part of the Mediterranean. Regarding the northern shore of the Mediterranean, distribution is more successful, with the entry of North Macedonia and Albania.

The Routes of the Olive Tree were decertified by the EPA Governing Board in 2025.

***Iter Vitis* Route**

The route involves countries such as France, Italy, Greece, Spain, Portugal, among others. Explore the culture of vineyard and wine, connecting historic wine regions from the Atlantic to the Caucasus, and from the Mediterranean to the Baltic ( Kharatishvili, 2023–2024). Archaeology has been the most developed area of knowledge with the work of this route, thanks to the study of the different containers used to store wine (examples from Crete, Lebanon and Rome). Wine is also highlighted as a common identity element of the Mediterranean, included in the Mediterranean diet, as well as its “cultural landscape” shared by the various countries of the region, a common denominator that thus connects them. They have also developed cooperation in advanced research, with the project “History Viticulture Oenology”, which studies the indigenous grape varieties of Sardinia. This research brings together the universities of Cagliari and Milan and has already identified “the remains of the oldest vine cultivation ever found in the western Mediterranean area”, belonging to the Nuragic civilization ( Dodd, 2019–2020).

**The Aeneas Route**

The Aeneas Route is a nautical route that stretches from the coast of Turkey to the Latium Vetus in Italy. The route is inspired by Virgil’s epic poem of Aeneas and aims to highlight the relationship between the historical and mythological culture of Asia Minor and the foundation of Europe. Based on the story, which took place between the shores of the Mediterranean, this route aims to recall the figure of Aeneas as a representation of the values of dialogue and tolerance among the peoples of the Mediterranean, empathy and solidarity. The route connects several archaeological sites recognized by UNESCO, such as Carthage, where it is intended to install a tourism dissemination plan, with a focus on digital, on the maritime adventure of Aeneas, recreating his journey through the various regions of the Mediterranean ( Yilmaz, 2020–2021).

**Via Francigena**

This route crosses the United Kingdom, France, Switzerland and Italy. It represents an ancient pilgrimage route that connects Canterbury (United Kingdom) to Rome and, later, to ports in southern Italy, connecting to the sea routes of the eastern Mediterranean. The 2023/24 report states that the integration of Santa Maria di Leuca (southern Italy) on the route is part of Euro-Mediterranean integration policies, thus contributing to intercultural and interreligious dialogue ( Gravari-Barbas, 2023–2024).

**European Route of Jewish Heritage**

The route crosses Spain, Italy, Greece, Turkey, among other countries. It includes Jewish communities and historical sites in Mediterranean countries, a sign of the diaspora and the Jewish presence in the Mediterranean over the centuries ( Borrione, 2018–2019).

**Roman Emperors and Danube Wine Route**

Serbia, Croatia, Bulgaria, Romania, etc., are some of the countries involved. Although centered on the Danube, this route extends to the Adriatic, integrating Roman sites in countries such as Croatia and Montenegro, and highlighting the Roman influence in the Mediterranean. By privileging the Roman heritage as the basis of European civilisation, it reinforces the common historical and cultural foundations that unite European countries ( Jovetic, 2022–2023).

**European Route of the Cistercian Abbeys**

The route includes countries such as Spain, Italy, Portugal, etc., thus bringing together abbeys located in Mediterranean regions. These places demonstrate the expansion of the Cistercian order and its influence on the cultural and religious landscape of the Mediterranean ( Beltramo, 2017–2018).

**Santiago de Compostela Pilgrim Routes**

Although traditionally associated with the Atlantic, some routes of the *Camino de Santiago* have branches that extend into the Mediterranean, especially through routes in Italy and southern France ( Robinson, 2023–2024).

Among them, five routes – the Phoenicians’ Route, the Routes of *El legado andalusí*, the Routes of the Olive Tree, the *Iter Vitis* Route, and the Aeneas Route – make explicit and consistent reference to Mediterranean cultural heritage. Their discourse is centred on themes such as intercultural dialogue, shared historical narratives, agricultural traditions, and mythological connections rooted in the Mediterranean basin. These routes demonstrate, both through documentation and concrete activities, that the Mediterranean is portrayed not only as a geographical area, but as a symbolic space for coexistence, mobility, and the foundation of a common European identity.

Conversely, five other routes – the *Via Francigena*, the European Route of Jewish Heritage, the Roman Emperors and Danube Wine Route, the European Route of the Cistercian Abbeys, and the Santiago de Compostela Pilgrim Routes – were included in the analysis due to their geographic presence in the Mediterranean area. However, their official discourse and narratives focus primarily on other cultural, spiritual or historical themes, and do not make direct reference to the Mediterranean as a cultural space. Nevertheless, as seen in the case of the *Via Francigena*, where the southernmost sites have recently been linked to Euro-Mediterranean cooperation policies, there is potential for this dimension to emerge in future strategic or interpretative developments across these routes.

Ultimately, the analysis invites further reflection on the diverse roles the Mediterranean plays – sometimes explicit, sometimes implicit – in shaping the narratives of cultural belonging within the framework of European identity.

## “Mediterranean Cultural Heritage” in academic research: identifying trends through bibliometric network analysis

To complement the previous analyses, we also explore how Mediterranean cultural heritage has been addressed in academic research over the past three decades. The analysis is based on a bibliometric study that includes publications from 1995 onwards, marking the beginning of the Barcelona Process – a key political initiative that sought to strengthen Euro-Mediterranean cooperation and cultural exchange. This chapter aims to highlight the dominant research trends, emerging priorities, and thematic gaps in the literature on Mediterranean cultural heritage, particularly in the context of European policy frameworks and scholarly engagement.

The data collection was conducted using Google Scholar, applying the search string: (“Mediterranean”) AND (“Cultural Heritage”) AND (“European”). The term “European” has been purposely included to identify publications that result from European initiatives, programs and funding.

The search was carried out through the Harzing’s Publish or Perish software, with a limit of up to 500 results. The resulting dataset was then processed and analysed using VOSviewer, a widely adopted tool for bibliometric mapping and visualization. After filtering, the analysis focused on 23 core items, enabling the identification of key thematic clusters, temporal patterns, and network structures in the academic discourse.

The bibliometric and visual analysis carried out using VOSviewer reveals a multidimensional research landscape that is both thematically rich and temporally dynamic. The Network Visualization serves as the central framework for understanding how different thematic areas are interconnected, organizing the keywords into distinct clusters that illustrate the main axes of discourse within this field.

**Figure 2.**
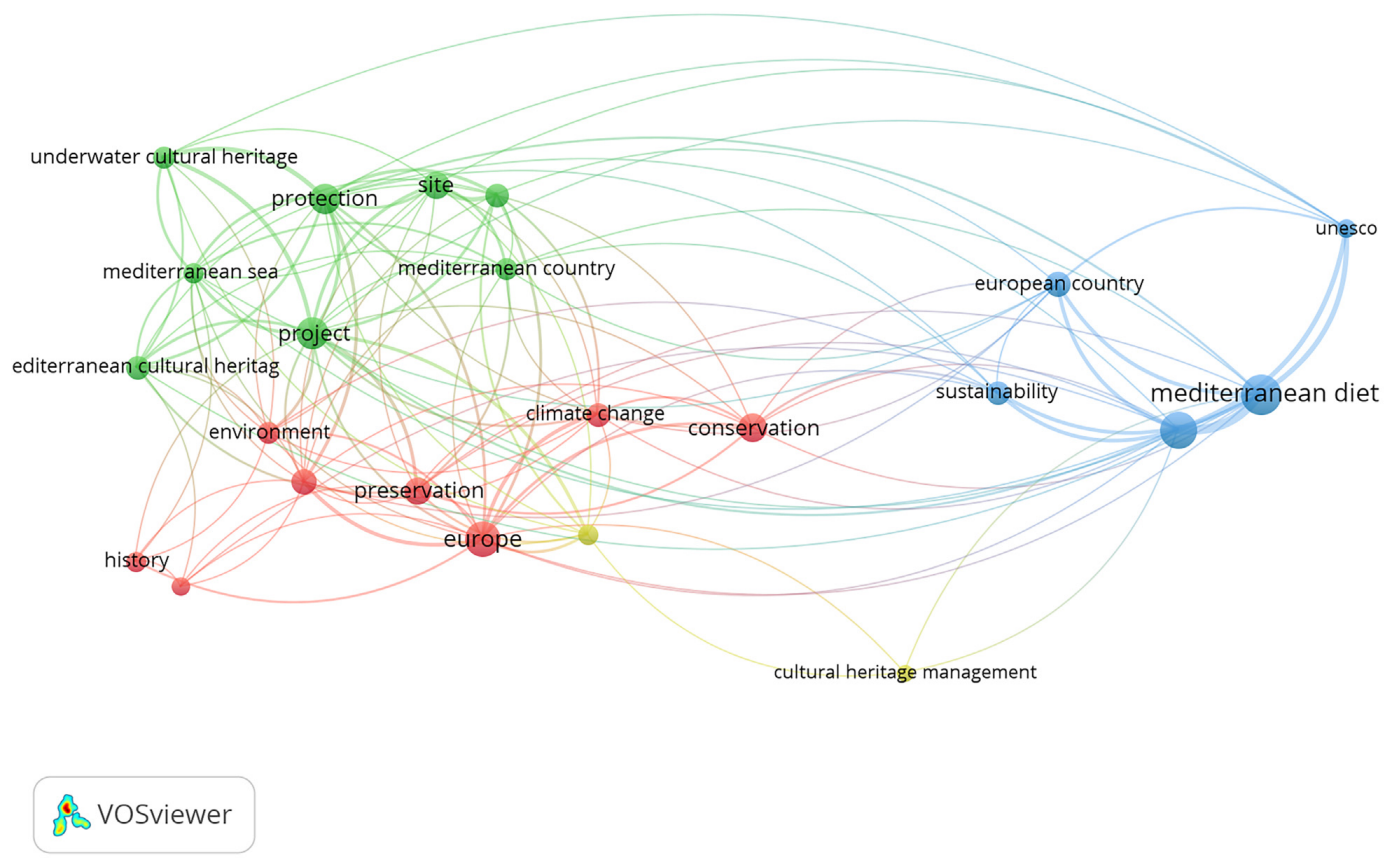
Network visualisation of clusters in publications on Mediterranean cultural heritage in VOSviewer.

In the Network Visualization, four prominent clusters are visible ( Figure 2).

The green cluster is focussed on concepts such as *protection*, *project*, *site*, *underwater cultural heritage*, *mediterranean sea* and *Mediterranean country*, indicating a strong focus on practical initiatives and localised efforts aimed at safeguarding *Mediterranean cultural heritage*. These nodes are tightly interconnected, reflecting how protection strategies are often operationalized through projects at specific cultural or natural heritage sites, including regional or macro-regional initiatives.

Adjacent to this, the red cluster brings together keywords like *climate change*, *preservation*, *conservation*, *environment*, *European identity* and *Europe*. This grouping highlights a thematic focus on environmental challenges, especially climate change, and their implications for the conservation of cultural heritage. The interconnection between *Europe* and *climate change* suggests that discussions about Mediterranean heritage are increasingly framed within broader European environmental and policy contexts.

On the opposite side of the map, the blue cluster focuses on *Mediterranean diet*, *intangible cultural heritage*, *sustainability*, *UNESCO*, and *European country*. This cluster reflects a different dimension of heritage: the intangible cultural practices and how these are embedded within sustainable living and global heritage frameworks promoted by organizations like UNESCO. The prominence and central position of the term *Mediterranean diet* indicate its pivotal role in recent academic and institutional discussions.

Finally, a yellow cluster, although smaller, represents the more technical and policy-oriented aspects of heritage studies, with *cultural heritage management* as its core, and directly linked to the keyword *European Commission*. This reflects specialized research dealing with governance, strategic planning, and institutional approaches to heritage preservation.

**Figure 3.**
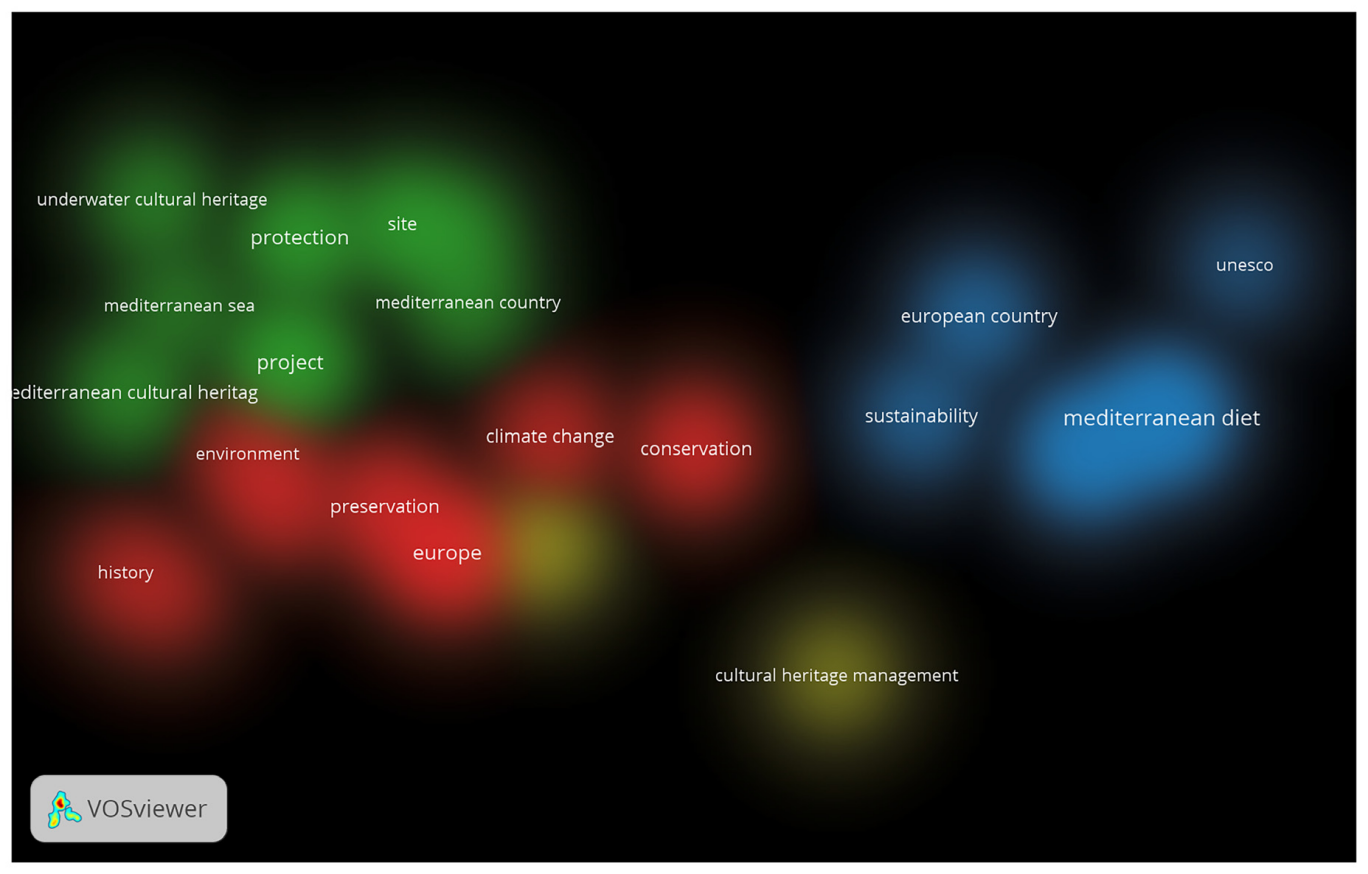
Density visualisation by clusters in publications on Mediterranean cultural heritage in VOSviewer.

These thematic groupings are reinforced and nuanced in the Density Visualization by Cluster, which visually amplifies the areas with greater keyword co-occurrence and thematic cohesion ( Figure 3).

The green and red clusters appear particularly dense, confirming the high volume of research and discussion on issues of protection and environmental threats. Similarly, the blue cluster's density underscores a growing academic interest in sustainable cultural practices and their recognition at the international level.

**Figure 4.**
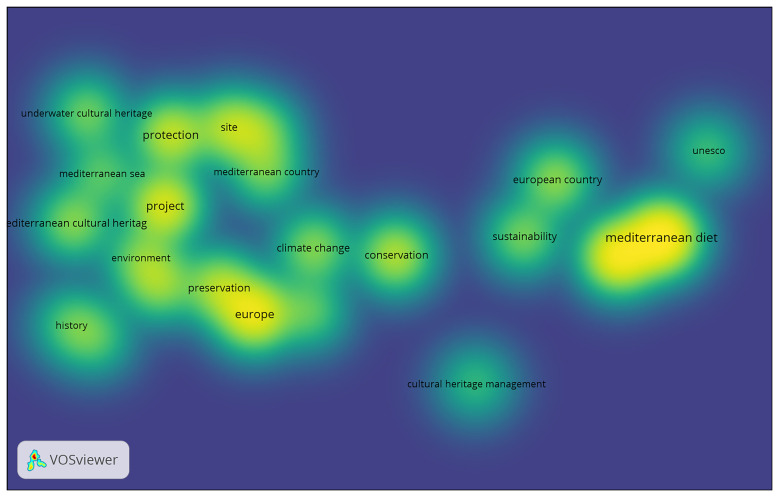
Density visualisation by items in publications on Mediterranean cultural heritage in VOSviewer.

Complementing this, the Density Visualization by Item reveals that terms like *Mediterranean diet*, *protection* and *preservation* are among the most frequently occurring and central in the dataset ( Figure 4). Their visual prominence confirms that these are the central themes around which much of the current scholarly discourse in *Europe* is structured. The presence of the keywords *UNESCO*, *project*, and *site* further illustrates how global frameworks and on-the-ground initiatives are being connected in the literature.

The analysis of the most cited publications reveals the works with the greatest impact and influence on the academic debate surrounding Mediterranean cultural heritage within the European context ( Table 2). There is a strong presence of interdisciplinary approaches, integrating themes such as sustainability, biodiversity, health, landscape planning, and traditional food practices.

**Table 2.** Top 10 most-cited publications on Mediterranean cultural heritage.

Author(s)	Year	Title	Journal name/Book Editor	Cites
Bach-Faig, A.; Berry, E. M.; Lairon, D.; Reguant J.; Trichopoulou, A.; Dernini, S.; Medina, F. X.; Battino, M.; Belahsen, R.; Miranda, G. & Serra-Majem, L.	2011	*Mediterranean diet pyramid today. Science and cultural updates.*	Public Health Nutrition	2431
Timothy, D.J.	2011	*Cultural heritage and tourism: An introduction*	Book: Channel View Publications	1461
Navarro, L.M. & Pereira, H. M.	2012	*Rewilding Abandoned Landscapes in Europe*	Book: Springer Open	1139
Blondel, J. ; Aronson, J. ; Bodiou, J-Y. & Bœuf, G.	2010	*The Mediterranean Region: Biological Diversity in Space and Time*	Book: Oxford University Press	1014
Camuffo, D.	2019	*Microclimate for cultural heritage: Measurement, risk assessment, conservation, restoration, and maintenance of indoor and outdoor monuments*	Book: Elsevier Science	996
Meskell, L.	2002	*Archaeology Under Fire: Nationalism, Politics and Heritage in the Eastern Mediterranean and Middle East*	Book: Routledge	758
Vos, W. & Meekes, H.	1999	*Trends in European cultural landscape development: Perspectives for a sustainable future*	Landscape and Urban Planning	712
González-Tejero, M. R.; Casares-Porcel, M.; Sánchez-Rojas, C. P.; Ramiro-Gutiérrez, J. M.; Molero-Mesa, J.; Pieroni, A.; Giusti, M. E.; Censorii, E.; de Pasquale, C.; Della, A.; Paraskeva-Hadijchambi, D.; Hadjichambis, A.; Houmani, Z.; El-Demerdash, M.; El-Zayat, M.; Hmamouchi, M. & Eljohrig, S.	2008	*Medicinal plants in the Mediterranean area: Synthesis of the results of the project Rubia.*	Journal of Ethnopharmacology	635
Bernués, A., Ruiz, R., Olaizola, A., Villalba, D., & Casasús, I.	2011	*Sustainability of pasture-based livestock farming systems in the European Mediterranean context: Synergies and trade-offs*	Livestock Science	528
Dernini S.; Berry E. M.; Serra-Majem L.; La Vecchia C.; Capone R.; Medina F. X.; Aranceta-Bartrina J.; Belahsen R.; Burlingame B.; Calabrese G.; Corella D.; Donini L. M.; Lairon D.; Meybeck A.; Pekcan A. G.; Piscopo S.; Yngve A. & Trichopoulou A.	2017	*Med Diet 4.0: The Mediterranean diet with four sustainable benefits*	Public Health Nutrition	517

The most cited article is *“Mediterranean diet pyramid today. Science and cultural updates”* by Bach-Faig *et al.* (2011), with 2,431 citations, which highlights the cultural, social, and nutritional benefits of the Mediterranean diet, framing it as a form of intangible heritage and sustainable practice. This topic is not only recurrent but also identified as one of the main clusters in thematic co-occurrence analyses.

Other highly cited publications include:

- *“Cultural Heritage and Tourism: An Introduction”* by D. J. Timothy (2011), with 1,461 citations, which explores the intersection between tourism and heritage, emphasising economic, identity-related, and conservation impacts.
- *“Rewilding Abandoned Landscapes in Europe”* by L. M. Navarro & H. M. Pereira (2012), with 1,139 citations, which links ecological and landscape restoration to the cultural and environmental value of rural Mediterranean territories.
- *“The Mediterranean Region: Biological Diversity in Space and Time”* by J. Blondel, J. Aronson, J.-Y. Bodiou & G. Bœuf (2010), with 1,014 citations, focusing on the ecological richness and historical development of the Mediterranean region.
- *“Microclimate for Cultural Heritage: Measurement, Risk Assessment, Conservation, Restoration, and Maintenance of Indoor and Outdoor Monuments”* by D. Camuffo (2019), with 996 citations, which addresses the impact of microclimatic conditions on the physical preservation of heritage monuments.
- *“Archaeology Under Fire: Nationalism, Politics and Heritage in the Eastern Mediterranean and Middle East”* by L. Meskell (2002), with 758 citations, which examines the tensions between heritage, nationalism, and politics in the eastern Mediterranean.
- *“Trends in European Cultural Landscape Development: Perspectives for a Sustainable Future”* by W. Vos & H. Meekes (1999), with 712 citations, analysing landscape transformation and proposing sustainable futures for European cultural landscapes.
- *“Medicinal Plants in the Mediterranean Area: Synthesis of the Results of the Project Rubia”* by M. R. González-Tejero *et al.* (2008), with 635 citations, which compiles ethnobotanical knowledge of traditional medicinal plants across Mediterranean countries.
- *“Sustainability of Pasture-Based Livestock Farming Systems in the European Mediterranean Context: Synergies and Trade-Offs”* by A. Bernués, R. Ruiz, A. Olaizola, D. Villalba & I. Casasús (2011), with 528 citations, assessing the social, ecological, and economic dimensions of traditional livestock systems.
- *“Med Diet 4.0: The Mediterranean Diet with Four Sustainable Benefits”* by S. Dernini *et al.* (2017), with 517 citations, which proposes an updated model of the Mediterranean diet, framed through four pillars of sustainability: health, environment, culture, and economy.

These data show that the academic debate on Mediterranean cultural heritage is deeply rooted in diverse research perspectives and enriched by a wide range of disciplines – from archaeology and nutrition to ecology, tourism, conservation, and spatial planning.

The five most prolific authors in the analysed dataset reflect the diversity of approaches in the study of Mediterranean cultural heritage, encompassing fields such as food policy, geospatial technology, preventive conservation, and European cultural studies.

S. Dernini – with 5 publications, is a leading figure in promoting the Mediterranean diet as cultural heritage, a healthy dietary model, and a sustainable food system. His work, published in journals such as *Public Health Nutrition*, *Frontiers in Nutrition*, and the *International Journal of Environmental Research and Public Health*, addresses food sustainability, public health, and intangible heritage. He is affiliated with the FAO (Food and Agriculture Organization of the United Nations) and CIHEAM Bari (International Centre for Advanced Mediterranean Agronomic Studies, Italy).

A. Agapiou – also with 5 publications, specialises in the application of remote sensing and GIS technologies to the management and monitoring of archaeological and cultural heritage sites. His research appears in journals like *Remote Sensing* ( Agapiou *et al.*, 2016) and *Heritage Science*, focusing on spatial analysis, photogrammetry, and change detection. He is affiliated with the Cyprus University of Technology.

V. Lysandrou – likewise with 5 publications, is a frequent collaborator of Agapiou and shares a technical focus on Earth observation and geospatial modelling applied to heritage conservation ( Lysandrou & Agapiou, 2016). His contributions span risk mapping, 3D documentation, and digital heritage, bridging the gap between data science, archaeology, and conservation technologies. He is also affiliated with the Cyprus University of Technology.

C. Sabbioni – with 4 publications, is a recognised expert in preventive conservation and the impacts of climate change on cultural materials. Her research combines climate data and scientific modelling with preservation and adaptation strategies ( Sabbioni *et al.*, 2010). She is affiliated with the National Research Council of Italy (CNR), specifically the Institute of Atmospheric Sciences and Climate, and has collaborated with institutions such as UNESCO and the European Commission.

T. Lähdesmäki – also with 4 publications, makes significant contributions to European heritage studies, cultural identity, and heritage policy. Her research explores EU institutional discourses on heritage and citizenship, focusing on the cultural dimension of political cohesion. She is a professor at the University of Jyväskylä, Finland, and actively involved in international projects on cultural memory and participatory heritage.

These affiliations show a strong presence of institutions located in the eastern and southern Mediterranean, particularly Cyprus and Italy, while also highlighting the relevance of northern Europe (Finland) in debates on cultural policy. The group includes scholars affiliated with key international bodies such as the FAO and UNESCO. Collectively, they represent the interdisciplinary, scientific, and international character of contemporary research on Mediterranean cultural heritage.

**Figure 5.**
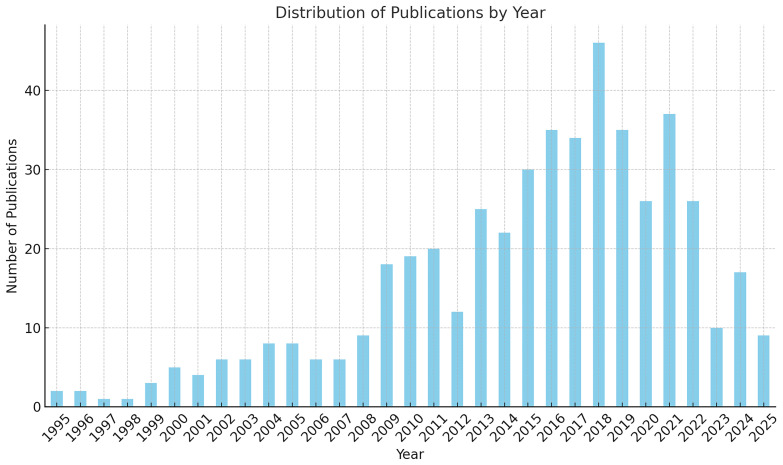
Distribution of publications by year on Mediterranean cultural heritage.

The distribution of publications by year shows a peak between 2016 and 2021, with more than 30 publications per year, reaching a maximum of 46 in 2018 ( Figure 5).

**Figure 6.**
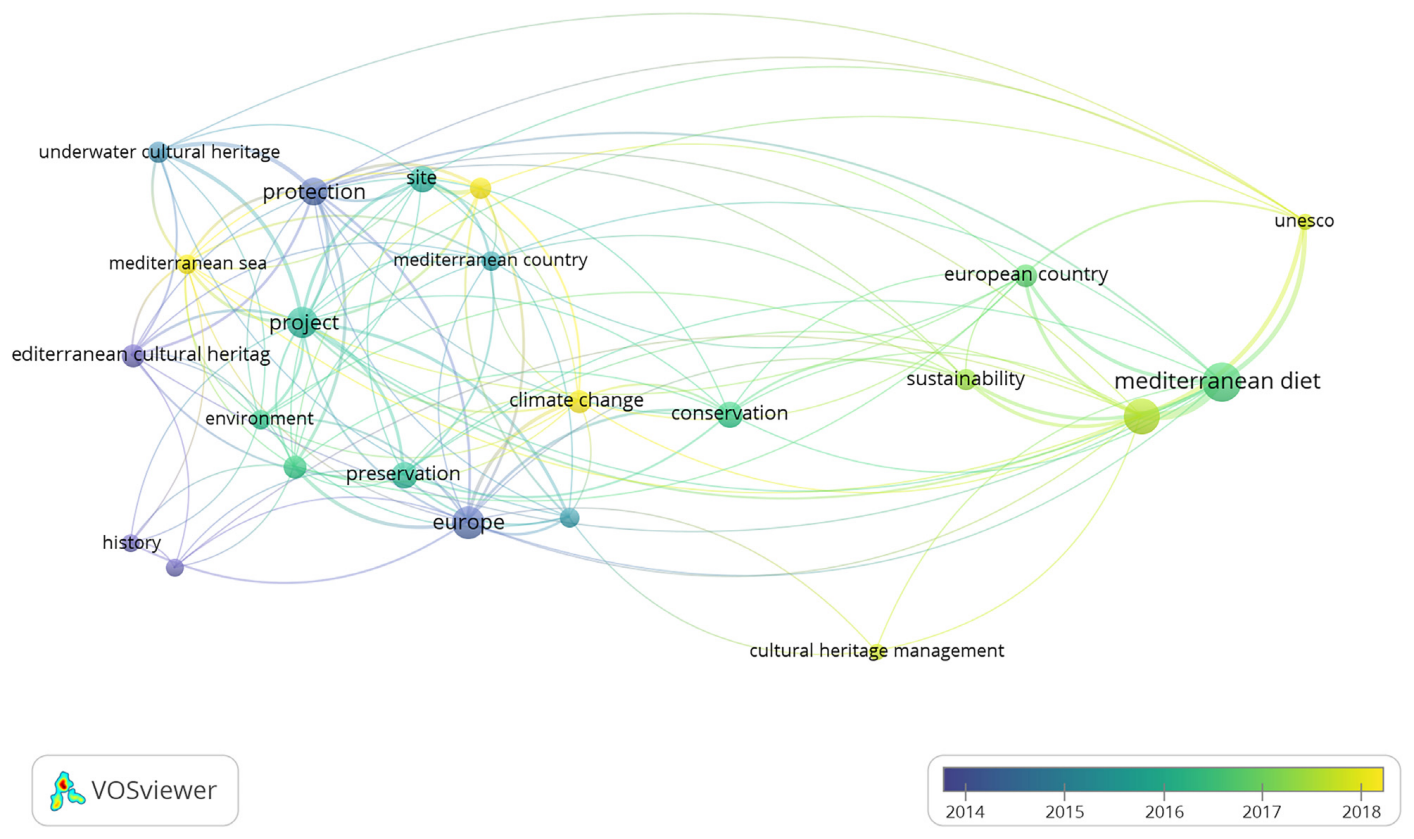
Overlay visualisation of publications on Mediterranean cultural heritage in VOSviewer.

Adding a temporal dimension for this period, the Overlay Visualization in VOSviewer allows us to see how the topics have evolved over time ( Figure 6).

Keywords shaded in lighter colours (yellow-green) represent more recent appearances in the literature. Terms like *Mediterranean diet*, *sustainability*, *climate change*, and *site* stand out as emerging or increasingly emphasized topics from 2016 onward. This shift reflects a growing awareness of how environmental sustainability and cultural heritage are interlinked, as well as a more contemporary focus on the intangible dimensions of heritage. Although the relationship between policy making and scientific production warrants further analysis – given that scientific output may also be influenced by other factors – we ventured some preliminary analyses, which we present in the following lines.

This evolution coincides with the development of key European strategic agendas, which help explain the growing research interest in topics such as the *Mediterranean diet*, *sustainability*, and *climate change*.

The recognition of the *Mediterranean diet* as Intangible Cultural Heritage by UNESCO in 2010, and its extension to additional countries in 2013, marked an important moment in the international validation of Mediterranean cultural practices, reinforcing their academic and political relevance. This growing recognition paved the way for further investigations into the intersections between intangible heritage and sustainability.

On the other hand, the European Year of Cultural Heritage (EYCH) in 2018 served as a significant catalyst for increased scholarly attention to cultural heritage topics across Europe. As is often the case with EU-designated thematic years, it also provided a stimulus for researchers and institutions to align their work with the year’s focus, fostering greater visibility and academic interest in cultural heritage.

The adoption of the United Nations' Sustainable Development Goals (SDGs) in 2015, which came into force in 2016, contributed to a broader global agenda that emphasized the importance of culture in sustainable development – particularly through Goal 11.4, which calls for strengthening efforts to protect and safeguard the world’s cultural and natural heritage. These developments provided an important framework that influenced European academic and institutional agendas in the years that followed.

Conversely, terms like *history*, *Europe*, and *Mediterranean cultural heritage* appear in darker shades (blue-purple), indicating that these have been present in earlier literature but may now serve more as contextual or foundational concepts rather than focal points of current research. This temporal layering demonstrates an evolution from general discussions on cultural heritage to more specialized and urgent topics like climate resilience and sustainable cultural practices.

In summary, the Network Visualization provides the structural backbone of the analysis, revealing how themes are grouped and interrelated. The Density Visualizations highlight where scholarly attention is most concentrated, while the Overlay Visualization illustrates how that focus has shifted over time. Together, these visual tools offer a coherent and multidimensional view of the research landscape on Mediterranean cultural heritage in a European context, showing that contemporary debates are increasingly shaped by environmental concerns, sustainability, and global heritage governance.

Among the publications from 2019 to 2025, in addition to sustainability, three thematic areas stand out for their growing relevance: climate change, digital technologies, and cultural tourism. Climate-related topics reflect a heightened interest in the relationship between cultural heritage and climate change. This includes research on the vulnerability of heritage sites, strategies for resilience, and the role of cultural heritage in fostering environmental awareness and contributing to sustainable development.

The rising focus on climate change-related heritage research aligns with strategic EU-level initiatives. Notably, the 2021 report *Strengthening Cultural Heritage Resilience for Climate Change*, produced by the Open Method of Coordination (OMC) expert group of the European Commission, explicitly calls for the integration of cultural heritage into climate and sustainability policies. The report recommends, among other measures, the development of a European climate risk map for heritage, knowledge-sharing platforms, and targeted investment in adaptation strategies. Additionally, the *European Cultural Heritage Green Paper*, also published in 2021, reinforces the role of cultural heritage within the European Green Deal and promotes its inclusion in climate action agendas. Digital heritage also emerges as a significant research area during this period. Many publications focus on the application of digital tools for the documentation, visualization, and interactive engagement with cultural heritage – particularly through technologies such as 3D modelling, augmented reality (AR), and virtual reality (VR). This trend aligns closely with broader European Union digitalization policies and reflects a growing emphasis on innovation in heritage preservation and accessibility. The European Commission has consistently encouraged Member States to enhance the digital transformation of the cultural sector. A major step in this direction was the launch of the *European Collaborative Cloud for Cultural Heritage (ECCCH)* in 2023, a digital infrastructure designed to connect cultural institutions and researchers across Europe, enabling collaboration and technological innovation in the heritage field.

Cultural tourism represents another prominent topic among the works published between 2019 and 2025. This renewed focus can be contextualized by earlier policy initiatives, notably the *Agenda for a Sustainable and Competitive European Tourism* introduced in 2007, which continues to influence research and strategic approaches within the sector. This agenda emphasized the importance of protecting cultural and natural resources through sustainable tourism models. The re-emergence of tourism as a research theme after 2019 may also be interpreted as a response to the redefinition of tourism strategies in the post-pandemic context, with a renewed focus on sustainability and heritage-led regeneration.

Taken together, these findings suggest a strong convergence between scholarly production and evolving European policy priorities. Researchers are not only responding to institutional frameworks but also anticipating and informing them – particularly in relation to climate resilience, digital innovation, and sustainable cultural tourism. This alignment points to the fact that cultural heritage is increasingly understood as a dynamic and strategic resource for supporting global strategies within the European context.

A regional gap is evident in the limited representation of the southern and eastern Mediterranean. While the term “Mediterranean” is widely used, sub-regions such as the Maghreb, Levant, and Middle East are largely absent. The dataset reflects a strong Eurocentric focus, with minimal inclusion of perspectives from non-European Mediterranean countries. Although the dataset in this case was limited by the inclusion of the term “European”, the fact remains that there is virtually no evidence of joint research between scholars from the northern and southern shores of the Mediterranean.

A closer examination of the 500-item dataset confirms this imbalance. Only five publications make explicit reference to countries in the southern Mediterranean – namely Morocco, Lebanon, Jordan, and Libya – and in each case, the authorship is confined to regional or external (often European) teams, without evidence of trans-Mediterranean co-authorship. Furthermore, no authors affiliated with North African or Middle Eastern institutions appear more than once in the dataset, and there is no recurring representation of southern Mediterranean scholars in this body of work. These findings reinforce the notion of a structural research gap, not only in terms of thematic focus but also in terms of academic collaboration and institutional presence.

This lack of scientific collaboration had already been noted by Kourtelis (2024).

Another significant gap lies in the limited attention to immaterial and community-based heritage. The analysis reveals a strong emphasis on monumental heritage and particularly on the Mediterranean diet, which alone appears in over 100 entries. However, more specific references to cultural practices, local festivities, oral traditions, or traditional knowledge systems across the Mediterranean are largely absent. Similarly, key terms such as *community*, *participation*, *living heritage*, and *co-creation* appear only occasionally, indicating that the social and participatory dimensions of heritage are still emerging within current academic discourse.

Nonetheless, while academic research increasingly aligns with broad European policy goals, there is still a noticeable lack of critical analysis regarding the specific impact of institutional instruments and initiatives. Despite some references to the European Union, the dataset reveals a lack of explicit connection with major cultural policy frameworks. Notably absent are mentions of tools such as the European Heritage Label (EHL), the Cultural Routes of the Council of Europe, or key heritage strategies promoted by the Union for the Mediterranean (UfM). The complete absence of references to the 2018 European Year of Cultural Heritage – whether in keywords or publication abstracts – is particularly striking, given its symbolic and strategic significance in promoting shared cultural values across Europe and the Mediterranean. This suggests that, despite general thematic alignment, there remains a disconnect between scholarly output and the critical evaluation of the concrete mechanisms through which cultural heritage policy is implemented – at least within the Euro-Mediterranean context examined here.

A more in-depth bibliometric analysis would be essential, particularly one that examines co-authorship networks, the role of leading institutions, and patterns of international collaboration. However, such an analysis was not feasible in the present study, as we deliberately chose not to use databases such as Scopus or Web of Science, where the limited results would not have provided a representative or reliable picture of the field.

Finally, it is worth noting, in the field of promoting scientific production and strengthening higher education in the region, the establishment of two Euro-Mediterranean universities.

In June 2008, the Euro-Mediterranean University (EMUNI) was inaugurated in Slovenia. EMUNI ( https://emuni.si/) is a university that operates in partnership with other universities, with a “network of collaborating higher education institutions from across the Mediterranean”. It is also a “platform for inter-cultural dialogue and science diplomacy”. The university's training offer includes master's degrees in digitalisation, human rights and intercultural dialogue. EMUNI also has a scientific journal, the “International Journal of Euro-Mediterranean Studies” (IJEMS), “an open-access peer-reviewed journal for the study of the Euro-Mediterranean region” ( https://emuni.si/publications/ijems/).

But the Euro-Mediterranean vocation of this institution is not an isolated case. We referred earlier to the Euro-Mediterranean University of Fes (UEMF), which was created later with the same values and ideals in mind ( https://ueuromed.org/).

## Conclusions

We are convinced that this study adds an additional layer to the analyses of the impact of Euro-Mediterranean policies and contributes directly to a deeper understanding of European policies for cultural heritage in the Mediterranean region and their real effects.

The state of the art presents a scenario in which the effectiveness and actual impact of Euro-Mediterranean initiatives in the field of cultural cooperation remain limited, facing several challenges and recurring criticisms. As highlighted by various authors cited, including Roger Albinyana, Jean-François Daguzan, and Damien Helly, although Euro-Mediterranean policies have evolved from fragmented economic agreements to more ambitious forms of cooperation, they continue to encounter serious obstacles: structural imbalances, difficulties in implementation, and persistent geopolitical tensions.

The most common criticisms point to a disconnect between discourse and practice, Eurocentrism in prevailing approaches, and insufficient involvement of civil society. What is needed is a more coherent, equitable, and context-sensitive approach, particularly towards the southern Mediterranean. Recognising the importance of culture and heritage as tools for promoting dialogue and mutual understanding, a more inclusive and culturally sensitive strategy is essential to revitalise Euro-Mediterranean relations.

Our conclusions broadly align with these assessments, although recent initiatives and outcomes – particularly over the past five years – indicate a growing recognition of the need to safeguard and promote cultural heritage within the framework of European cultural policy and cultural diplomacy in the Mediterranean.

In the field of Euro-Mediterranean cooperation, the Union for the Mediterranean (UfM) appears increasingly committed to fostering cultural dialogue and promoting Mediterranean cultural heritage. This is reflected in initiatives such as Mediterranean Day, celebrated since 2021, and the launch of the Mediterranean Capitals of Culture and Dialogue in 2023, first held in 2025. These developments have opened the way for greater involvement of civil society, local communities, and regional institutions.

Regarding cultural cohesion and intra-European cooperation, the most recent monitoring report of the European Heritage Label (2024) raises concerns about how European significance is being addressed, communicated, and understood. The report suggests the creation of thematic and regional networks to strengthen cooperation and contribute to Europe’s cultural integration, highlighting the Mediterranean as a possible pilot example of a regional network initiated by the sites themselves. Opportunities for greater initiative from the sites and their communities are further supported by the establishment of the Bureau (2023) and the launch of the first dedicated funding call for EHL sites in 2025.

There is scope to further enhance the recognition of Mediterranean cultural heritage as a means of promoting both European and Mediterranean identity, while at the same time strengthening internal cohesion and external cooperation. The main challenge lies in shifting from a top-down approach – often characterised by the political instrumentalisation of heritage and sometimes imposed on communities, as in the case of the heritage-making of the Mediterranean Diet – to a bottom-up model in which processes actively involve Mediterranean citizens.

In terms of scientific research and production, several observations stand out. It should be noted, however, that this study was limited to articles and books written in English, thereby excluding important languages in the Mediterranean context such as French, Italian, Spanish, and even Arabic.

Scientific cooperation between European researchers and those from the southern Mediterranean remains weak, with scholarly output dominated by European authors and institutions. Moreover, European funding does not appear to be strongly reflected in the region’s research production. Bibliometric analysis shows that publications resulting from EU-funded projects are underrepresented, accounting for only 12% of the total, with no sign of growth in the past five years. This limited impact may be explained by the nature of the funded projects, which have tended to prioritise capacity building for professionals, heritage valorisation, and the involvement of civil society partners and policymakers, as seen for instance in programmes such as Euromed Heritage, Erasmus+, and Interreg, rather than pure academic research.

Overall, these findings point to the relatively limited impact of cooperation initiatives and project funding policies on cultural heritage research in the region. There is therefore considerable room for improvement and a clear need to strengthen scientific cooperation.

Such a path could be pursued in the context of an increasingly “Europe of the Regions”. Cultural heritage can play a key role in fostering inter-regional and trans-regional cooperation in the Mediterranean, both within the European Union and with its southern neighbours. This may be achieved through collaboration in areas such as cross-border heritage, maritime and underwater heritage, religious heritage, archaeological heritage, and intangible cultural heritage, as well as through partnerships among museum networks across the region.

There is, indeed, enormous potential for different nations in the Euro-Mediterranean space to build effective dialogue in the fields of research and public policy for the protection and valorisation of cultural heritage. Joint action is essential in addressing shared challenges, such as global warming, water scarcity, and rising sea levels that threaten cultural heritage along the Mediterranean coast, to name but a few.

The cultural heritage of the Mediterranean, as a fundamental pillar of Europe’s identity and diversity, deserves greater prominence in European cultural programmes. Its preservation and enhancement require such recognition.

Europeana, the EU’s digital platform for cultural heritage, illustrates how Mediterranean heritage can be integrated into the digital society. By making millions of works and documents freely accessible online – many of them from Mediterranean institutions – it guarantees wider visibility, open access, and long-term preservation. Mediterranean heritage is particularly well represented, with 23,763 results from a simple search for the term “Mediterranean” ( www.europeana.eu). This demonstrates both the richness of the region’s heritage and the growing importance of digital infrastructures in ensuring its transmission to future generations.

Taken together, these findings highlight both the achievements to date and the pressing need for more inclusive, collaborative, and culturally sensitive approaches if Mediterranean cultural heritage is to play its full role in shaping Euro-Mediterranean relations.

## Ethics and consent

Ethical approval and consent were not required

## Data Availability

Not Applicable **Copyright:** © 2025 Bombico and Garcia. This is an open access work distributed under the terms of the Creative Commons Attribution License, which permits unrestricted use, distribution, and reproduction in any medium, provided the original work is properly cited.
