# Supplementary material for: European identity and cultural heritage: the Mediterranean has its say
Source: Open Res Eur. 2026 Jan 13;5:321. Originally published 2025 Oct 17. [Version 2] doi: 10.12688/openreseurope.21507.2 (PMC12856248; doi:10.12688/openreseurope.21507.2)
Supplement: Peer review history

# Reviewer report: Reviewer response for version 2

**DOI:** 10.21956/openreseurope.24480.r68612

**Published:** 2026-01-29

**Authors:** Eszter György

**Affiliations:** 1 Eötvös Loránd University, Budapest, Hungary

Overall, this is a very interesting study, whose strength lies in its examination of the relationship between the Mediterranean region and European identity and cultural heritage across many different scales and fields of study. However, since the different fields and layers of study (the Mediterranean diet on UNESCO's list of intangible cultural heritage, various political and cultural advocacy/representative organizations and their local practices, such as cultural routes, the English-language academic publications related to the region) require very different methods and approaches, the style and the critical approach of the study also becomes uneven in places. Some parts of the study (such as the subchapter on the instrumentalization of heritage itself) are much more elaborate and reveal deeper critical observations than other sections (such as the one on cultural routes in the Mediterranean region), which seem more like short lists in a tourist brochure.

 Below, I will quote a few passages where, in my opinion, these inequalities are most evident.

*„The Mediterranean, therefore, emerges not only as a geopolitical space but as a vibrant cultural ecosystem, capable of fostering a renewed, resilient, and plural Euro-Mediterranean identity. Over the past decade, UfM’s actions consistently engage cultural heritage as a platform for cooperation, resilience, and identity-building, focusing on shared values, historical awareness, sustainable development, and the unifying power of culture in times of crisis.”* This conclusion does not seem critical enough, especially in light of the fact that the authors write in detail at the beginning of the study about the problematic and tension-filled construction of the Mediterranean as a concept. This summary sounds rather like a promotional material that ignores the previously mentioned criticisms about the understanding and interpretation of the Mediterranean region as a unified area.

 Concerning the sub-section about the cultural routes, the description of the Phoenician route in my opinion contains some errors that make the paragraph difficult to understand.

*“However, although this route sees the number of its partners increasing from year to year, the network faces difficulties on the southern shore of the Mediterranean, a key region for the construction of the main objective of the route – intercultural dialogue (* *Guérin, 2021–2022). On the other hand, the activities carried out within the framework of this route have mobilized countries such as Spain and Italy, which have organized meetings entitled “Euro-Mediterranean Dialogues”, between researchers studying the battle of Baecula (Spain) and the battle of Metaurus (Italy), as well as exhibitions on Hannibal's expedition, in Capua, Cretone (Italy). On the other bank, and despite the difficulties indicated above, dialogue partnerships have been established between members of this route and public institutions in Jordan, Palestine and Lebanon.”* What does “on the other bank” mean here and what are the precise difficulties indicated? They are not explained sufficiently in detail and therefore, it is not clear what the main problems are with this cultural route. Furthermore, if all the cultural routes in the region are listed, it would have been beneficial to have some minimal comparison, analysis, and joint interpretation beyond a simple list. Thus, reading about ten routes does not really contribute to a more complex understanding of the topic; it might have been enough to mention the most important ones and write more about them.

 The following subchapter, which contains the bibliometric analysis, is much more critical and analytical. Here, the list of the most important and most frequently cited articles and authors is nicely complemented by an interpretation of the topics and possible explanations for the emphases and shortcomings.  However, some parts of the conclusion, such as the sentences: *“The cultural heritage of the Mediterranean, as a fundamental pillar of Europe’s identity and diversity, deserves greater prominence in European cultural programmes. Its preservation and enhancement require such recognition.”* are again superficial and general. Moreover, what is also missing from the conclusion is the statement of a more general stake of the article. What did the authors want to suggest in reality by analyzing the impact of Euro-Mediterranean policies for cultural heritage? That the Mediterranean heritage is still underrepresented ( *“cultural heritage of the Mediterranean, as a fundamental pillar of Europe’s identity and diversity, deserves greater prominence in European cultural programmes.”* Was this conclusion reached in comparison with other European regions? The study itself does not necessarily reflect this, given the number of publications, cultural routes, and former cultural capitals in the region. Furthermore, given the number of heritage sites and intangible cultural heritage practices in the region, especially in Italy and Spain, it is difficult to believe that the Mediterranean region is "at a disadvantage" compared to the representation of Central and Eastern Europe or even the Balkans.

If any, are all the source data and materials underlying the results available?

Yes

Is the work original in terms of material and argument?

Yes

Is the work clearly and cogently presented?

Partly

Is the argument persuasive and supported by evidence?

Yes

Does the research article contribute to the cultural, historical, social understanding of the field?

Yes

Does it sufficiently engage with relevant methodologies and secondary literature on the topic?

Yes

Reviewer Expertise:

Cultural heritage studies, Minority heritage, Roma heritage and Roma social and cultural history

I confirm that I have read this submission and believe that I have an appropriate level of expertise to confirm that it is of an acceptable scientific standard, however I have significant reservations, as outlined above.

# Reviewer report: Reviewer response for version 1

**DOI:** 10.21956/openreseurope.23263.r63120

**Published:** 2025-12-24

**Authors:** Oscar Julian Esteban-Cantillo

**Affiliations:** 1 Institut Jean Nicod, Paris, France

This paper offers a well-structured and insightful analysis of how Mediterranean Cultural Heritage is mobilised within European identity-building and policy frameworks. I appreciate the combination of qualitative policy analysis with bibliometric methods, which provides a clear picture of both institutional narratives and academic trends over the last three decades. The authors show convincingly that the Mediterranean is increasingly used as a symbolic resource for cultural diplomacy, cooperation, and sustainability, even if often implicitly.

 However, the study also highlights important limitations: persistent Eurocentrism, uneven participation of southern Mediterranean countries, and the instrumentalisation of heritage for political purposes. These points are crucial and could have been explored even more critically, especially regarding power asymmetries in heritage making.

 I believe it is pertinent for the article to include a section referring to the methods used. These must be precise and clear to ensure that the research can be replicated in other areas of the world. Specify how the review was conducted, what criteria were taken into account, the period in which the review was conducted, and how many articles were considered. Then specify how the bibliometric analysis was performed, the clusters, the distances between nodes, and so on. This should be specified separately in the methodology. 

 Overall, the article succeeds in demonstrating that Mediterranean heritage plays a growing role in European identity strategies, while also calling for more inclusive, participatory, and context-sensitive approaches. It is a solid contribution that exposes both the potential and contradictions of Mediterranean cultural heritage within EU narratives.

If any, are all the source data and materials underlying the results available?

Yes

Is the work original in terms of material and argument?

Yes

Is the work clearly and cogently presented?

Yes

Is the argument persuasive and supported by evidence?

Yes

Does the research article contribute to the cultural, historical, social understanding of the field?

Yes

Does it sufficiently engage with relevant methodologies and secondary literature on the topic?

No

Reviewer Expertise:

climate change, conservation, cultural heritage, biodiversity

I confirm that I have read this submission and believe that I have an appropriate level of expertise to confirm that it is of an acceptable scientific standard, however I have significant reservations, as outlined above.

# Response

**Published:** 2025-12-29

**Authors:** Sónia Bombico

We would like to thank you for the review and to inform you that we have submitted a second version that incorporates the improvements you suggested.

# Reviewer report: Reviewer response for version 1

**DOI:** 10.21956/openreseurope.23263.r63119

**Published:** 2025-11-03

**Authors:** Rumpapak Luekveerawattana

**Affiliations:** 1 Suan Sunandha Rajabhat University, Bangkok, Thailand

**1. Originality:**

 The work is **moderately to highly original**, especially in linking Mediterranean Cultural Heritage (MCH) with European identity through both policy and bibliometric analysis. While the theoretical framing draws on established heritage studies, the **regional synthesis and empirical scope** are new and valuable.

**2. Engagement with Methodology and Literature:**

 The study shows **strong engagement with secondary literature** on Mediterranean history and heritage policy but **limited engagement with qualitative methodological frameworks**. It would benefit from clearer explanation of data selection, analytical procedures, and reference to qualitative research methods.

**3. Clarity and Cogency:**

 The paper is **well-structured and intellectually coherent**, though occasionally dense and verbose. The argument develops logically but could use **sharper transitions** and more explicit research questions to improve readability.

**4. Persuasiveness and Evidence:**

 The argument is **largely persuasive and supported by extensive documentary evidence**. However, the link between data and interpretation could be clearer, and inclusion of contrasting perspectives would strengthen the overall case.

**5. Data Availability:**

 Only **partially available**. The policy documents used are public, but the underlying bibliometric data are not provided, limiting full transparency.

If any, are all the source data and materials underlying the results available?

Partly

Is the work original in terms of material and argument?

Yes

Is the work clearly and cogently presented?

Yes

Is the argument persuasive and supported by evidence?

Yes

Does the research article contribute to the cultural, historical, social understanding of the field?

Yes

Does it sufficiently engage with relevant methodologies and secondary literature on the topic?

Partly

Reviewer Expertise:

Tourism management

I confirm that I have read this submission and believe that I have an appropriate level of expertise to confirm that it is of an acceptable scientific standard.

# Response

**Published:** 2025-12-29

**Authors:** Sónia Bombico

We would like to thank you for the review and to inform you that we have submitted a second version that addresses some of the observations you raised.
